# Key Applications and Potential Limitations of Ionic Liquid Membranes in the Gas Separation Process of CO_2_, CH_4_, N_2_, H_2_ or Mixtures of These Gases from Various Gas Streams

**DOI:** 10.3390/molecules25184274

**Published:** 2020-09-18

**Authors:** Salma Elhenawy, Majeda Khraisheh, Fares AlMomani, Mohamed Hassan

**Affiliations:** 1 Department of Chemical Engineering, College of Engineering, Qatar University, Doha P.O. Box 2713, Qatar; se1105821@student.qu.edu.qa (S.E.); falmomani@qu.edu.qa (F.A.); 2 Center for Advanced Materials, Qatar University, Doha P.O. Box 2713, Qatar; mohamed.hassan@qu.edu.qa

**Keywords:** membrane separation, selectivity, permeability, SILMs, ILPMs, ILMMMs, gas separation

## Abstract

Heightened levels of carbon dioxide (CO_2_) and other greenhouse gases (GHGs) have prompted research into techniques for their capture and separation, including membrane separation, chemical looping, and cryogenic distillation. Ionic liquids, due to their negligible vapour pressure, thermal stability, and broad electrochemical stability have expanded their application in gas separations. This work provides an overview of the recent developments and applications of ionic liquid membranes (ILMs) for gas separation by focusing on the separation of carbon dioxide (CO_2_), methane (CH_4_), nitrogen (N_2_), hydrogen (H_2_)_,_ or mixtures of these gases from various gas streams. The three general types of ILMs, such as supported ionic liquid membranes (SILMs), ionic liquid polymeric membranes (ILPMs), and ionic liquid mixed-matrix membranes (ILMMMs) for the separation of various mixed gas systems, are discussed in detail. Furthermore, issues, challenges, computational studies and future perspectives for ILMs are also considered. The results of the analysis show that SILMs, ILPMs, and the ILMMs are very promising membranes that have great potential in gas separation processes. They offer a wide range of permeabilities and selectivities for CO_2_, CH_4_, N_2_, H_2_ or mixtures of these gases. In addition, a comparison was made based on the selectivity and permeability of SILMs, ILPMs, and ILMMMs for CO_2_/CH_4_ separation based on a Robeson’s upper bound curves.

## 1. Introduction

Flue gas emissions from the chemical industries have severe impacts on the whole environment and the atmosphere and are considered one of the primary causes of global warming phenomena [1,2,3,4,5,6,7,8]. Consequently, the separation and the capture of these unwanted gases has become necessary. The unwanted gaseous emissions that result from burning fuel and chemicals in chemical engineering power plants include carbon dioxide (CO_2_), methane (CH_4_), nitrogen oxides (NOx), sulphur oxides (SOx), and hydrogen sulphide (H_2_S). Each power plant has the responsibility of mitigating and reducing the amount of these gases to limit their negative impact on the environment. 

**Figure 1:**
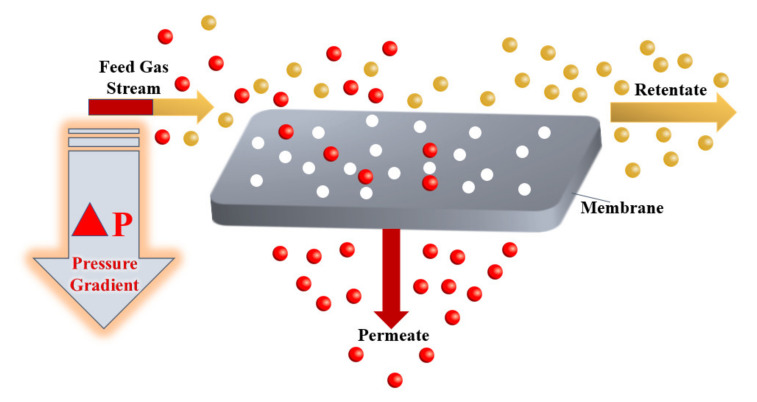
Theoretical scheme of the membrane gas separation process.

The gas separation process plays a crucial and a fundamental role in the chemical engineering industry as a result of its broad range of applications, ranging from carbon dioxide gas separation from natural gas [9,10,11], recovery of hydrogen from waste gas streams [12], production of nitrogen and oxygen-enriched gases. In the past decades, a large number of different methods for the separation of gases have been developed, including membrane separation [13], chemical looping [14,15,16,17] and cryogenic distillation [18,19,20,21]. The gas separation process using membrane technology depends on the sorption-diffusion mechanism or sieving [22]. Commercial membranes for gas separation use membranes that are nonporous and asymmetric based on a solution-diffusion transport mechanism, and both vapour and gas separation are based on the same mechanism [13]. At the heart of a gas separation process, the driving force for the separation of gases is a pressure gradient. A high-pressure gas mixture moves across a membrane surface that has a selective permeability to one component of the gas mixture. Hence, the permeate produced as a result of this mechanism is enriched with this specific component [23]. Figure 1 below shows a schematic representation of the membrane gas separation process.

In the meantime, there is a growing interest in more sustainable gas-separation processes that are mainly based on selective gas absorption or adsorption. Ionic liquids (ILs) provide the best alternative to most of the conventional liquids used in gas separation processes. The unique properties of these ionic liquids distinguish them from all the liquids used in the separation processes [24]. Incorporating ionic liquids into membranes (ILMs) generally increases the membrane selectivity towards specific gases, the permeability of gases through it and the mass transfer. There are three main classifications of ionic liquid membranes (ILMs). These classifications are supported ionic liquid membranes (SILMs), ionic liquid polymeric membranes (ILPMs), and ionic liquid mixed-matrix membranes (ILMMMs) [25]. Each type of ILM has different applications in gas separation processes. The applications of the SILMs, ILPMs, and ionic liquid mixed-matrix membranes (ILMMMs) in the gas separation processes of CO_2_, CH_4_, N_2_, H_2_ from various gas streams are discussed thoroughly through this paper. Table 1, Table 2 and Table 3 below shows the names and abbreviations of the principal ionic liquid membranes, primary gases, ionic liquids, and polymers mentioned in this study.

## 2. Global Warming

**Figure 2:**
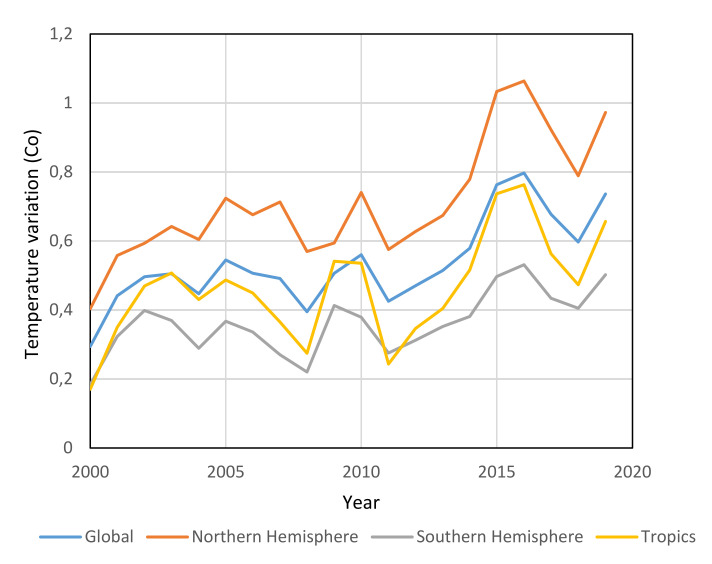
Median temperature variations at different locations [26].

The topic of global warming has been the focus of research around the world. Global warming is a continuous rise in the planet Earth’s average temperature with time (Figure 2). This increase in the average temperature is due to the greenhouse effect (Supplementary Materials Figure S2). The greenhouse effect is an occurring process that happens when gases in the Earth’s atmosphere trap the radiations coming from the Sun. Figure 2 and Figure S1 shows how the temperature has increased at different locations with time as a result of global warming. 

It is seen from Figure 2 that there is a general increasing trend in the temperature variations in all of the specified locations with time. The Northern hemisphere has faced the highest increase in its climate temperature, reaching a 1.064 °C increase in the year 2016. This increment in the climate indicates that the baseline temperature of the Northern hemisphere has increased by 1.064 °C. On the other side, the Southern hemisphere has faced the least temperature increase. This difference between the two hemispheres temperatures can be owed to the ocean circulation patterns, specifically the North Atlantic Oscillation. Thus, resulting in higher variations in temperature of the climate in the northern hemisphere.

One of the sources that can be used to get insights about the interest of the researchers on different topics from all over the world in different periods is google trends. The sample size used in research must be large enough, in order to get the most accurate sample statistics that approximates the population parameters. Google processes around 70000 search queries in 1 s, 5.8 billion searches per day, and approximately 2 trillion global searches per year. Consequently, Google trends were used in several figures in this review paper.

As a result of the devastating impacts caused by global warming, several publications have been made to study these impacts and how to limit these adverse effects. Looking at Figure S1 which shows the worldwide search interest trend of the global warming topic in Google, the numbers on the y-axis represent the search interest in the topic of global warming relative to the highest point on the chart for the given time. The value of 100 is the peak popularity for the term. Also, a value of 50 means that the term is half as popular as 100. A score of 0 shows that there are not enough data for this term. It is obvious that the highest search interest in global warming was in the year 2007 with a peak of 100 and the lowest search interest is in the year 2020. Perhaps the year 2020 has the lowest search interest in global warming because most of the people are mainly focusing on the COVID-19 pandemic disease topic. Furthermore, the graph shows a general decreasing trend in the search interest as time increases.

Figure 2 presents the worldwide search interest trend of the greenhouse gases topic in Google. It is clear that that the highest search interest in the greenhouse gases a 100-value peak was in the year 2007. Hence, Figures S1 and S2 show that greenhouse gases are directly linked to global warming.

The primary greenhouse gases in the atmosphere are carbon dioxide (CO_2_) and methane (CH_4_). Carbon dioxide is released in the atmosphere through several natural and anthropogenic sources. The chemical industry is considered one of the primary anthropogenic sources of CO_2_ in the atmosphere. In the chemical industries, the fossil fuels containing the hydrocarbons are burned for several purposes releasing CO_2_. Consequently, after the industrial revolution, the concentration of CO_2_ has increased by more than a third. Figure S3 shows the global CO_2_ concentration (ppm) in the atmosphere.

Figure S3, which shows the global CO_2_ concentration (ppm) in the atmosphere, illustrates that the concentration of CO_2_ has a steep increase with time. Also, the relationship between the concentration of CO_2_ and time is a strong positive linear relationship with a coefficient of correlation (R^2^) = 0.9975. These results places the atmosphere of the Earth at very high risk, and the industries must try their best to decrease their emissions of CO_2_. Figure S4 and Table S2 shows the CO_2_ emissions in tones from gaseous processes in several Arab countries. It is evident that Saudi Arabia is the country that has the highest emissions of CO_2_ from the year 2000 to the year 2018. In the year 2018, Saudi Arabia has emitted 187,089,100 tonnes of CO_2_ to the atmosphere. These high emissions of CO_2_ are due to the high number of gas processes used in the country. On the contrary, Jordan is ranked the lowest CO_2_ emitter among this group. In the year 2018, Jordan has emitted 6,883,700 tonnes of CO_2_ to the atmosphere from gas processes, which is almost 27 times less than the emissions in Saudi Arabia. As a result of these significant emissions of CO_2_ from the gas processes. Researchers, engineers, and chemists are trying to find ways in order to decrease these emissions. One of these ways is by using gas separation processes at which CO_2_ will be separated from a various gas stream. Figure S5 shows how the interest of the people worldwide varies with time in the CO_2_ gas separation process. By looking at Figure S5, it can be concluded that many people across the world are interested in the topic of CO_2_ gas separation. There are several peaks with a value of 100 in this graph; this means that this topic is of high importance and is usually considered. 

Furthermore, from the most gases produced in the combustion process in the chemical industries is the greenhouse gas methane (CH_4_). In the burning of fossil fuels containing the hydrocarbons, methane is released in the flue gas and into the atmosphere. This fuel-burning process has increased the concentration of methane dramatically in the atmosphere. Figure S6 shows how the global concentration of methane in the atmosphere changes with time.

It can be concluded from Figure S6 that the global methane (CH_4_) gas concentration in the atmosphere (ppb) has a steep increasing trend with time. These scary concentrations knock on the emergency doors of all chemical industries.

## 3. Ionic Liquid Membranes (ILMs) in Gas Separation Processes

As a result of global warming and the harmful effects of greenhouse gases on the whole environment, the gas separation process plays a fundamental role in the chemical engineering industry as a result of its broad range of applications ranging from carbon dioxide gas separation from natural gas, Recovery of hydrogen from waste gas streams, production of nitrogen and oxygen-enriched gases. During the past decades, a large number of different techniques for the separation of gas have been developed and these methods include membrane separation, chemical looping, and cryogenic distillation. By focusing on the membrane gas separation, many studies and publications were made by several researchers from all over the globe to study the membrane gas separation processes synthesis, implementations, applications, limitations, and development. Figures S7 and S8 give an insight into the interest of researchers about the gas separation processes from the year 2004 to the year 2020.

By looking at Figures S7 and S8, it is seen that there is a considerable interest in gas separation processes in all over the world. In the year 2004, a maximum peak (100) was reached. Furthermore, the country that shows the most considerable interest in membrane gas separation is Malaysia (27%). This means that 27% of the total searches made in Google on the gas separation process topic were made by researchers from Malaysia, which is quite a high percentage since there are approximately 2 trillion global Google searches made per year. The second highest region is South Korea (26%), which is a pretty high percentage. By focusing on the membrane gas separation, there are several types and classes of membranes used across the industries. One of the best classes of membranes to be used in the gas separation processes are ionic liquid membranes (ILMs). The incorporation of the ionic liquids into membranes (ILMs) generally increases the membrane selectivity towards specific gases, the permeability of gases through it, and mass transfer. There are three main classifications of the ionic liquid membranes (ILMs). These classifications are supported ionic liquid membranes (SILMs), ionic liquid polymeric membranes (ILPMs), and ionic liquid mixed-matrix membranes (ILMMMs). Each type of ILM has different applications in the gas separation processes.

### 3.1. Ionic Liquids

Ionic liquids (ILs) are organic molten salts that have a melting point below 100 °C. Room temperature ionic liquid (RTIL) are ionic liquids that have a melting point less than room temperature (T_melting_ < 373 K) [27]. These ionic liquids exhibit many exciting properties that distinguish them from other liquids, including negligible vapour pressures, high thermal stability [28,29,30], inflammability, a liquid range of up to at least 300 °C [31], high solubility for a wide range of inorganic and organic compounds [32]. Moreover, their physicochemical properties can be tailored to satisfy specific chemical tasks by the appropriate selection of anion, cation, and substituents on the cationic constituent. Table 4 below shows the two- and three-dimensional structures of the most used ionic liquids mentioned in this study.

It is obvious that from Figure S9 that ionic liquids are capturing the special attention of researchers from various countries across the globe. China is ranked the highest country (12%) by searches and interest in ionic liquids, followed by South Korea (9%). Consequently, extensive chemical engineering applications of these innovative fluids have taken place by the end of the last century [33,34,35,36,37,38]. Ionic liquids are made by associating large organic cations with a numerous variety of anions. The most common ionic liquid cations include pyridinium, imidazolium, pyrrolidinium, and theirmono or poly-alkyl derivatives, as well as tetraalkylammonium or phosphonium and trialkylsulfonium. The classical ionic liquids anions include, for the low melting ionic liquids: trifluoromethylsulfate (TfO), bis(trifluoromethylsulfonyl)imide (NTf_2_), tetra-fluoroborate (BF_4_) or hexafluorophosphate (PF_6_) and dicyanamide (N(CN)_2_). In low stability ionic liquids or ionic liquids that are not liquid at room temperature, the following simple anions are included: iodide, nitrate, bromide, chloride, perchlorate, formate or acetate [39]. Ionic liquids can be produced by other anions, as shown by the selective alkyl-methylimidazolium IL list presented in Table 5 below.

Aside from their different properties, there are severe limitations that face the usage of ionic liquids [40,41]. The main limitations are the high synthesis price and the high recycling energy requirement. These two limitations could act as a burden on the economic and financial sides of the processes. The use of ionic liquid membrane (ILM) technology can overcome these two drawbacks. The ILM consists of the feed and permeates phases that are separated by a membrane containing IL that allows simultaneous extraction and stripping at each side of ILM. The IL for an ILM can be stabilized by either quasi-solidification to endow material with good mechanical strength or impregnating it inside the pores of the support membrane. The ILM techniques require a fewer amount of IL as a carrier and do not require additional steps for IL recycling. Consequently, ILMs have many advantages, such as the low energy requirements, the ease of fabrication due to the flexible and compact devices, low operating costs, capital and several more merits

Furthermore, due to the special and the outstanding properties of IL, e.g., high viscosity, and negligible vapour pressure, ILMs are considered more stable compared to the traditional supported liquid membranes (SLMs) based on organic solvents. Therefore, ILMs show promising application potential. In the last decade, researchers have reported the use of ILMs in several applications and fields, including the separation of various mixtures, electrochemical devices, and catalytic reactions.

The research progress of gas separation, especially CO_2_ separation, has been addressed in depth. Martins, et al. [42] conducted a CO_2_ removal process by using a membrane contactor that is combined with a biocompatible ionic liquid (IL), cholinium lysinate, that has a high absorption capacity (5.9 mol CO_2_/kg IL). The rate of CO_2_ removal and IL solution regeneration was examined in their study by changing the feed gas composition, conditions of the ionic liquid flow rate, and the relative humidity. The results obtained from their study have shown that the proposed system is capable of removing CO_2_ from anaesthetic gas circuits [42]. Shamair, et al. [43] have successfully investigated the performance of a recently synthesized RTIL in SILM, theoretically, and experimentally. In their study, benzimidazolium-1-acetate was used to synthesize SILM over a polyimide support. The synthesized SILM was tested for different gas mixtures (CO_2_/CH_4_ and CO_2_/N_2_). The prepared SILM results have shown that there is a high selectivity of 37.92 and 40.29 for CO_2_/CH_4_ and CO_2_/N_2_, respectively. These results strengthen the DFT calculation and indicate that there are up-and-coming applications for SILM in gas separation [43]. Sohaib, et al. [44] have mentioned in their research study that coupled absorption/desorption in combination with ILs, can be considered very useful for continuous post-combustion carbon capture [44]. Zia-ul-Mustafa, et al. [45] focused their research on the effect of imidazolium-based ionic liquids on the PES membrane for CO_2_/CH_4_ separation. The polyethersulfone (PES) based gas separation ionic liquid polymeric membranes (ILPMs) were synthesized in their study. Ionic liquids 1-ethyl-3-methylimidazolium tetrafluoroborate [EMIM][BF_4_] and 1-ethyl-3-methylimidazolium dicyanamide [EMIM][DCA] were added to the doping solution at ten weight % and flat sheet dense membranes were cast by the method of dry phase inversion. The CO_2_ gas permeability of the synthesized ILPMs was successfully increased by blending the ionic liquids into the polymer matrix as a result of the high affinity and solubility of CO_2_ in [EMIM][BF_4_] and [EMIM][DCA] ionic liquids. Membrane PES + IL1 (10%) has shown an approximation of 23.5 folds increase in CO_2_ gas permeability in comparison to the pure polyethersulfone membrane. Hence, they mentioned that the ILPMs have a remarkable potential for the separation of CO_2_ from natural gas [45]. Nabais, et al. [46] used poly (ionic liquid)-based engineered mixed matrix membranes for the separation of CO_2_/H_2_. The mixed matrix membranes (MMMs) were synthesized by combining IL, a pyrrolidinium-based PIL, and three highly CO_2_-selective metal-organic frameworks (MOFs). The different MOFs ZIF-8, (MIL-53(Al), and Cu_3_ (BTC) _2_ were used as fillers, that aims to maximize the performance of the membranes towards the purification of syngas. Ideal selectivity and permeability of CO_2_ were achieved as a result of their usage of different MOFs and loadings (0, 10, 20, and 30 wt %). The authors also mentioned that this has caused mechanical and thermal stabilities of the membranes and has in term increased their performance [46].

#### 3.1.1. Selected Physicochemical Properties of Ionic Liquids

Ionic liquids are very versatile and their anions and cations can be adapted easily to the role that they have to play. Their most valuable and distinguishable physicochemical properties include the following: low melting point (m.p. < 100 °C), high thermal stability, insignificant vapour pressure, a low surface tension [47], high electrical conductivity, a wide electrochemical window [48,49,50], and an adjustable solvent viscosity and/or hydrophobicity and/ or polarity associated with high or low water miscibility [51]. In the separation techniques, the most crucial parameters are the ionic liquid melting point, viscosity, thermal stability, vapour pressure and solvent properties [52].

#### 3.1.2. Melting Point

Extensive research has been made to understand the low melting points of the ILs and to predict their physicochemical properties by using computational tools. By using a large number of ILS that have been studied, Table 5 above shows the selected physicochemical properties of methylimidazolium ionic liquids only [39]. Low melting points are obtained with salts made of large and asymmetrical ions [50]. Generally speaking, the melting temperature T_m_ decreases by increasing anisotropy, internal flexibility, and size of the ions. On the other side, the melting point increases by increasing the interactions of the alkyl chains [53]. The change in the melting points of the ionic liquids is linked to the symmetry or cation size, as the chain length changes the melting point changes. Nasirpour, et al. [54] researched the relationship between chain length and the melting point by examining the Ionic liquids melting point versus the length of the alkyl chain for [C_n_mim][PF_6_] and trihexyl-alkylphosphonium hexafluorophosphate [P666n][PF_6_]). The results of their study have shown that the alkylmethylimidazolium [PF_6_] ionic liquids have shown a decrease in the melting temperature as the alkyl chain length increase. Shorter alkyl chains with *n* < 6 have higher melting points since they produce symmetrical Ionic Liquids with better ion cohesion. Also, longer alkyl chains with *n* > 8 have higher melting points since their cations are more hydrophobic and form Van der Waals forces [52,54].

#### 3.1.3. Viscosity

Ionic Liquids at room temperature are viscous liquids. By looking at Table 5, at 25 °C the lowest viscosities observed with the methylimidazolium salts are 15 cp for [EMIM][C(CN)_3_], 16 cp for [EMIM][N(CN)_2_], and 20 cP for [allylmim][N(CN)_2_]. [55]. The values of these viscosities are similar to the viscosities at 25 °C of viscous organic solvents such as ethylene glycol (16 cP) [56], dimethyl phthalate (14 cP) [57], or ethanolamine (21 cP) [58]. An increase in temperature dramatically reduces the viscosity, as shown by the 50 °C values listed in Table 5. There is an extensive database in the literature that includes the viscosities of ionic liquids [59].

#### 3.1.4. Vapour Pressure and Thermal Stability

The significant vapour pressure of the ILS is quite challenging to be measured. At high temperatures, a decomposition of the liquids might occur, and at very low temperatures the vapour pressure becomes too low to be measured by typical conventional methods. Several studies focus on finding the vapour pressure of ionic liquids. Ahrenberg, et al. [60] and his research team have focused on determining the vapor pressure of ionic liquids at low temperatures using AC-chip-calorimetry. A highly sensitive method for mass loss determination at temperatures starting from 350 K was successfully developed in their study. They found the vapour pressure from the measured rates of mass loss using the Langmuir equation. The method that they have used has successfully determined the vapour pressure and the vaporization enthalpy of an archetypical ionic liquid 1-ethyl-3-methylimidazolium bis (trifluoromethylsulfonyl) imide ([EMIM][NTf_2_]) [60]. Maton, et al. [61] have mentioned in their research that the thermal stability of an IL depends on the specific combination of the cation-anion [61]. Also, Xue, et al. [62] have examined the thermal stability of ILs under vacuum by thermogravimetric analysis (TGA). The TGA analysis of their study has shown that many ionic liquids start to decompose thermally at 700 K [62].

### 3.2. Supported Ionic Liquid Membranes (SILMs)

**Figure 3:**
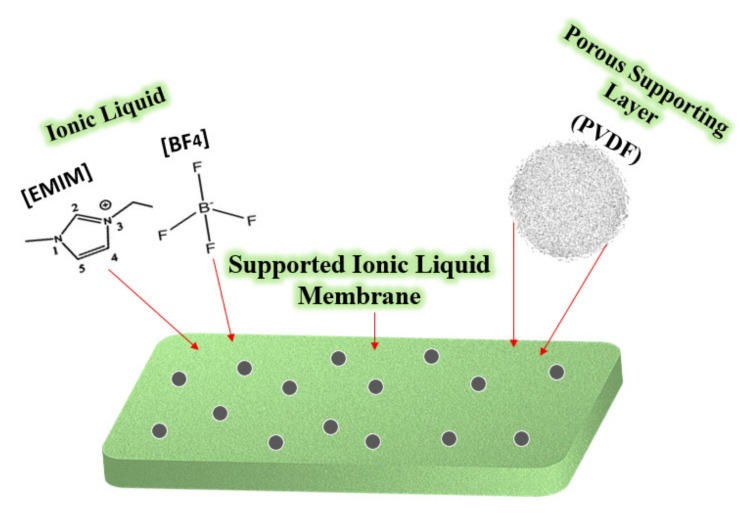
Theoretical scheme of a supported ionic liquid membrane (SILM).

The emissions of carbon dioxide (CO_2_) and other acid gases like SO_2_, H_2_S, and CH_4_ have to be efficiently controlled due to the devastating harm and impacts that they produce on the whole environment and society. A fascinating approach for acid gas removal or separation from other gases is by using supported ionic liquid membranes (SILMs). Ionic liquids (ILs) are very promising compounds for this application as a result of their very low or almost negligible vapour pressure, which leads to high stability. This, in terms, reduces the solvent losses by the volatilization into the gas stream. The selection of the separation process technique is a very critical and fundamental issue in the chemical industries. Among a variety of conventional methods for the separation processes, supported Ionic liquid membranes (SILM)-based separation processes have been predicted as a very promising option that has a very bright future in the gas separation industry. Figure 3 below shows a schematic representation of supported ionic liquid membrane (SILM).

**Figure 4:**
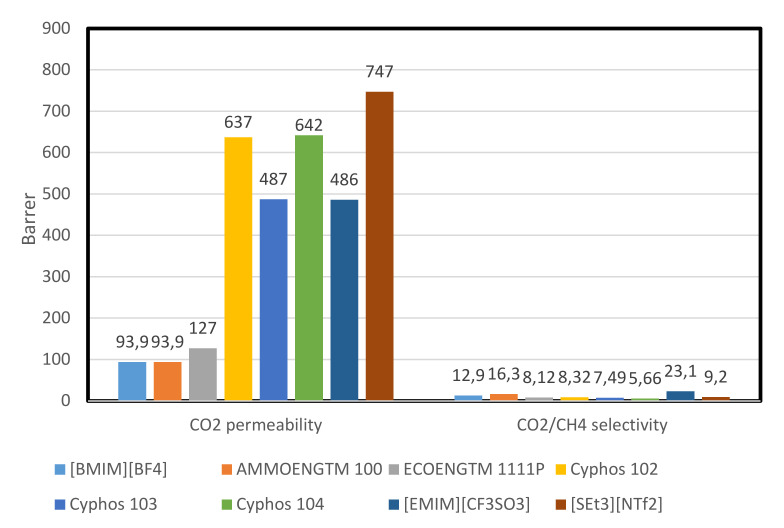
CO_2_ permeability and CO_2_/CH4 selectivity of some SILMs [63].

The SILM has shown a high selectivity for some industrially essential gas pairs, e.g., CO_2_/N_2_, CO_2_/CH_4_, and SO_2_/CH_4_. SILMs assures to provide the more stable form of membranes, that has the minimum liquid loss because of its ILs high viscosity and the increased capillary forces between the membrane support and the ionic liquid [13]. Figure 4 below shows the CO_2_ permeability and CO_2_/CH_4_ selectivity of some SILMs.

Figure 4 shows the CO_2_ permeability and CO_2_/CH_4_ selectivity of some SILMs. The highest CO_2_ permeability is shown by [SEt_3_][NTf_2_] (747 bar), while the highest CO_2_/CH_4_ selectivity with a value of 23.1 is shown by [EMIM][CF_3_SO_3_]

There is a lot of research and work readily available in the literature in which SILMs were deeply studied for potential applications in the gas separation process. The first research about gas separation using SILM dates back to the year 1995. In this first research, Quinn, et al. [64] have prepared new facilitated transport membranes and examined them under various conditions. These membranes can selectively permeate CO_2_ from hydrogen and methane. Their membranes were made up of the salt hydrates tetramethylammonium fluoride tetrahydrate ([(CH_3_)_4_N]F·4H_2_O), or tetraethylammonium acetate tetrahydrate([(C_2_H_5_)_4_N]CH_3_CO_2_·4H_2_O), immobilized in films of Celgard 3401^®^. The results of their study shows that at the operating temperature of 50 °C, both membranes had exhibited CO_2_ permeability. The CO_2_ permeability has increased with the decrease of the partial feed pressure of CO_2_. Besides, the selectivities of CO_2_/CH_4_ and CO_2_/H_2_ had increased with the decrease of the feed.

On the other hand, the selectivities of CO_2_/H_2_ were low because of the permeation of H_2_ through the Celgard^®^ dense phase. The modelling of their membrane properties was successfully consistent with the CO_2_ permeation using a mechanism of facilitated transport with reasonably derived diffusivities for the chemically bound CO_2_ carrier species [64]. Scovazzo, et al. [65] examined the CO_2_ separation from N_2_ using RTILs with and without ionic and neutral doping compounds. The authors have presented a proof-of-concept of SILMs, the basic principles of FILM development, and have also discussed the future needs for continuing the development of SILMs and FILMs. The results of their research have shown that SILM had a CO_2_ permeability of 4.6 × 10^-11^ mol/(cm^2^ kPa s) with selectivity over the air of 29; those values are competitive with existing membrane materials. Also, the FILMs had a 1.8 improvement in the CO_2_ permeability with a driving force of 4.6 kPa of CO_2_ [65]. Also, Scovazzo [66] has further researched on proving that the supported ionic liquid membranes (SILMs) outperform the standard polymers for the separations of CO_2_/N_2_ and CO_2_/CH_4_, even under continuous flow mixed gas conditions. The analysis of his research has predicted a maximum CO_2_-permeability for SILMs and an upper bound for permeability selectivity vs CO_2_-permeability with respect to the CO_2_/N_2_ and CO_2_/CH_4_ separations. A massive data for the Methane and inorganic gas permeabilities and selectivities at 30 °C for different RTILs are provided in Table 6 [66].

Because of the improved CO_2_ capture performance at low concentration, the potentialities of novel technology of SILMs for the absorption process in the gas-liquid membrane contactor system are being extensively explored in the meantime to improve and complement previous technology. Ramli, et al. [67] have focused their research on the application of membrane contactors in a combination of monoethanolamine as a solvent system for post-combustion CO_2_ capture. In their research, a modified hydrophobic gas-liquid membrane contactor system was synthesized using 1-ethyl-3-methylimidazolium bis (trifluoromethylsulfonyl)imide [EMIM][NTf_2_] ionic liquid as a supporting phase and Liqui-Cel^®^ parallel flow module as membrane support. By using the synthesized modified module and under moderate operating conditions, parallel flow mode, and by using monoethanolamine (MEA) as an absorbent, the effects of absorbent temperature and gas velocity on the CO_2_ absorption efficiency and CO_2_/O_2_ selectivity were successfully determined. For further analysis, the performances of the blank and the modified membrane module were implemented and compared at different temperatures (303–348 K) and gas velocities (4.63×10^−6^ to 3.70×10^−5^ m s^−1^). The results of their study have shown that the CO_2_ absorption efficiency of the modified module is approximately doubled with an average selectivity factor of CO_2_/O_2_ around five times compared to the blank contactor system. Consequently, their modified membrane contactor system had shown great potential for further usage in the industrial process of CO_2_ capture [67]. As time passes, Shamair, Habib, Gilani and Khan [43] has examined the performance of a newly synthesized RTIL in SILM, experimentally, and theoretically. Benzimidazolium-1-acetate was used in his study to synthesize SILM over polyimide support. The synthesized SILM was tested for different gas mixtures (CO_2_/N_2_ and CO_2_/CH_4_). The membranes were tested under different operating conditions in order to accurately analyze the commercial potential of these membranes. The Density functional theory (DFT) calculations were also made in order to predict the interactions of CO_2_ with the RTIL. The authors’ study results shown that SILM has high selectivity of 37.92 and 40.29 for CO_2_/CH_4_ and CO_2_/N_2,_ respectively. The authors also made an Arrhenius plot of permeability and selectivity of CO_2_/CH_4_ to analyze the temperature change effect on selectivity and permeability of CO_2_/CH_4_ at a temperature range of 25–65 °C. Their Arrhenius plot has shown that the permeability of CO_2_ is directly proportional to the temperature and the selectivity of CO_2_/CH_4_ is inversely proportional to the temperature. The Arrhenius behaviour was observed in the CO_2_ permeability since the coefficient of determination (R^2^) is 0.9872. These observations proof the interaction between CO_2_ and RTIL. This relation can be attributed to the increased diffusion rate of CO_2_ in the membrane. It can be concluded from the Shamair, Habib, Gilani and Khan [43] study that the increased diffusion rate can be as a result of the decreased viscosity of the RTIL as the temperature is increased. This decrease in viscosity leads to a higher molar free volume for the gas to pass through the membrane matrix. In contrast, the solubility was decreased as a result of the reduced interaction between CO_2_ and RTIL. In addition, the solubility was decreased because the viscosity of the RTIL has decreased as the temperature increases, which allows more diffusion of gases resulting in a loss of the selectivity of CO_2_/CH_4_. Also, the leap down of selectivity can be linked to better CH_4_ permeability, because of the increased free volume. Thus, based on the study’s Arrhenius plot, as the temperature increases, the permeability of CO_2_ increases, and the selectivity of CO_2_/CH_4_ decreases [43]. Schott, et al. [68] have tested five new RTILs as a supported ionic liquid membrane (SILMs) for CO_2_/N_2_ separation capability. This new series of ILs contains bicyclic amidine cations of 1,8-diazabicyclo[5.4.0]undec-7-ene (DBU) or 1,5-diazabicyclo[4.3.0]non-5-ene (DBN) with small alkyl chain substituents. These Ionic liquids have been prepared in their study in order to investigate the effects of the cation structure on the free volume. In addition, relevant chemical and physical properties have been examined that includes CO_2_ viscosity and solubility. Also, each cation was paired with two different anions: tetracyanoborate and bis(trifluoromethanesulfonyl)imide, for further investigation of the cation-anion interaction on the SILM selectivity and permeability. The results of their study have shown that three of these new Ionic liquids have exceeded the Robeson’s upper bound for CO_2_/N_2_ gas separations [68].

Due to the wide applications of supported ionic liquid membranes (SILMs) in gas separation and purification, especially for CO_2_ capture, this topic captures the eye of many researchers at which they focus all of their effort in introducing new methods and approaches in order to increase the selectivity, permeability, and performance of the SILMs. Mohammadi, et al. [69] have examined a new molecular level approach in order to analyze different types of ILs for the use in SILMs and thereby to evaluate the performance of the membrane by using quantum molecular chemical modeling and calculations. The authors have developed several relationships for the selectivity and the permeability of SILMs. Also, they validated the relationships using a collected experimental data of the relevant gas pairs in the separation applications. For the comparison purpose and the evaluation of the model performance, they have used the accumulative absolute relative deviation (AARD (%)). The results of their research have shown that the proposed approach has provided an accurate, extendable, pure predictive, and reproducible method for the estimations of the SILMs performance [69]. Karousos, et al. [70] have investigated the N_2_ and CO_2_ mixed-gas permeation rates through a supported ionic liquid membrane (SILM), which was developed on a ceramic composite nanofiltration substrate with a 10 nm separation layer pore size. The ionic liquid used as a supported liquid phase was 1-methyl-3-octylimidazolium tricyanomethanide. A cyclic heating process was used to subject the hybrid membrane to different temperatures that include isothermal steps of 180 °C, and under inert gas flow. Hence, the temperature effect and the feed composition effect on the CO_2_/N_2_ separation performance and the permeation properties were studied before and after the end of each heating cycle. The results of the authors study have shown that, for all the heating cycles, the largest CO_2_/N_2_ selectivity values were determined at ambient temperature. Furthermore, the thermal behavior of the IL phase was affected by the confinement into the nanopores of the synthesized SILM which has also caused decomposition of the IL phase within the heat treatment process. In addition, some functionalized by-products were detected by the UV/Vis spectroscopy as a result of prolonged periods of heating at 180 °C. The formation of these by-products can lead to the regeneration of a stable form of chemical complexes with CO_2_ molecules, which can dramatically affect the solubility and diffusivity of CO_2_, and the separation efficiency of CO_2_/N_2_ [70].

The removal of the acid gases from natural gas has captured the attention of several researchers, and many studies were made on them. Park, et al. [71] have used a multi-phase separation process incorporated with RTILs and polymer. In their study, a new SILMs were synthesized for acidic gas removal from crude natural gas. The polymer materials used for the membrane are polyvinylidene fluoride (PVDF), and the RTILs used are [Bmim][BF_4_]. To investigate the permeation properties of acid gases, SILMs were examined for CO_2_, CH_4_, and H_2_S permeation at different operating conditions. The results of their investigation show that the permeability coefficients of CO_2_ and H_2_S gases were found to be high at 30–180 and 160–1100 bar, respectively. Also, their selectivity values were 25–45 for CO_2_/CH_4_ and 130–260 for H_2_S/CH_4_ [71]. Santos, et al. [72] prepared supported ionic liquid membranes (SILMs) with the use of acetate-based room temperature ionic liquids (RTILs) for the selective separation of carbon dioxide (CO_2_) from nitrogen (N_2_) using 1-butyl-3-methylimidazolium acetate ([BMIM][Ac]), 1-ethyl-3-methylimidazolium acetate (EMIM][Ac]), and the monomer vinylbenzyl trimethylammonium acetate ([Vbtma][Ac]). The room temperature ionic liquids were supported in a porous membrane of polyvinylidene fluoride and at a temperature range of 298–333 K, several permeation experiments were performed. The results of their experiments have shown that the permeability of the gases increases as the temperature increases. On the other hand, the selectivity of CO_2_/N_2_ decreases as the temperature increases for all of the RTILs studies [72]. Luis, et al. [73] determined the removal of CO_2_ and SO_2_, the permeabilities of CO_2_, air, and 10 vol. % SO_2_–air and the selectivities of CO_2_ and SO_2_ mixture by using various SILMs. The results of their study have shown that air permeability is one order of magnitude less than the permeability of CO_2_ and it is less than the permeability of the air mixture and 10 vol.% SO_2_ [73]. Liu, et al. [74] studied the effect of using different ILs in the SILMs on the separation of CO_2_/H_2_and CO_2_/N_2_ gas mixtures. In their study, four supported ionic liquid membranes (SILMs) were synthesized by fixing the following ILs: 1-butyl-3-methylimidazolium trifluoromethanesulfonate ([BMIM][TfO]), 1-butyl-3-methylimidazolium bis((trifluoromethyl)sulfonyl)imide ([BMIM][NTf_2_]), 1-butyl-3-methylimidazolium dicyanamide ([BMIM][DCA]), and 1-butyl-3-methylimidazolium acetate ([BMIM][AC]) in a polyvinylidene fluoride (PVDF) membrane. The results of the authors’ experiments have shown that the SILMs rate of gas permeation increases with the temperature increase. The best separation performance of CO_2_/H_2_ was shown by [BMIM][TfO] SILM with permselectivity of 16.2 and CO_2_ permeability 40 °C of 1966 bar. Moreover, the best separation efficiency for CO_2_/N_2_ was shown by [BMIM][AC] SILM with permselectivity of 21.8 and CO_2_ permeability at 30 °C of 520 bar [74].

Some limitations face the use of the ILS for the CO_2_ separation process in the industrial and academic fields; among these limitations are the high price and the toxicity level of the ILs. These two limitations have limited the application and the design of the SILMs. Fan, et al. [75] addressed this issue by preparing SILMs with ILs that have a green and cost-effective characterization. In their study, [choline] [pro]/polyethylene glycol 200 (PEG200) mixtures were used to synthesize novel SILMs. Also, CO_2_/N_2_ separation was examined at a temperature range of 308.15 to 343.15 K. The results of their experiments have shown that the CO_2_ permeability was increased by a factor of 3 as the viscosity of the liquids has decreased from 370 to 38 mPa·s. Overall resistance was also decreased, and SILMs process has changed from diffusion-control to reaction-control addition as a result of adding PEG200 [75].

SILMs are considered very promising media for the natural gas separation process. However, most research focuses on the selective separation of CH_4_ and CO_2_ while paying minimal attention to selective separation in SILMs of (H_2_S and CO_2_), and (H_2_S and CH_4_). Zhang, et al. [76] examined the permeability of CO_2_, CH_4_, and H_2_S in two different categories of ILs (basic and neutral ionic liquids) under dry conditions. The ideal selectivities of H_2_S/CO_2_, CO_2_/CH_4_, and H_2_S/CH_4_ were calculated. The results of their study shows that the neutral ILs 1-butyl-3-methylimidazolium trifluoro-methanesulfonate) and (1-butyl-3-methylimidazolium tetrafluoroborate) show a high CO_2_ and H_2_S permeability and a competitive permselectivity of CO_2_/CH_4_ and H_2_S/CH_4_. Consequently, they can be used for the simultaneous removal of CO_2_ and H_2_S from natural gas. Also, the basic ILs (1-butyl-3-methylimidazolium acetate) has shown a high permeability of H_2_S and CO_2_, and an extremely high permselectivity of H_2_S/CO_2_, CO_2_/CH_4_, and H_2_S/CH_4_. Hence, they can be successfully used for the selective removal of H2S from natural gas. In addition, facilitated transport of CO_2_ and H_2_S were reported in 1-butyl-3-methylimidazolium acetate [76]. Close, et al. [77] synthesized SILMs by impregnating porous anodic alumina membranes with seven different IL, with trifluoroacetate anions, different alkyl imidazolium cations, bis (trifluoromethylsulfonyl) imide, and acetate. The results of their study shows that the permeability of CO_2_ yielded more than 1000 bar and ideal selectivities of CO_2_/N_2_ were within 12 and 21. Also, the permeance values of the 100 nm nominal pore size support membranes were higher than the permeance values of the 20 nm nominal pore size support membranes [77].

In the selective separation of CO_2_ from flue gas, a new pathway for the design of novel CO_2_ separation materials was proposed. Zhang, et al. [78] designed and synthesized a series of imidazolium-based phenolate ILs with dual-site interaction centres for the isolation of CO_2_ from N_2_ by the SILMs. Under humidified conditions, 1-butyl-3-methylimidazolium phenolate ([BMIM][PhO]) containing 15 wt % H_2_O has achieved a high selectivity of CO_2_/N_2_ (reaching 127) and a permeability (reaching to 2540 bar). Also, a novel facilitated mechanism of transport in which CO_2_ is transferred from carbene to phenolated anion is proposed by the authors based on the results of the theoretical calculations, FT-IR and NMR [78]. Hojniak, et al. [79] synthesized and incorporated imidazolium, piperidinium, pyrrolidinium, and morpholinium ILs with a side chain containing triethylene glycol and tosylate anions, and their symmetrical dicationic analogs into the SILMs. The results of their research have shown that the selectivities of the dicationic ionic liquids are greater than the values of the corresponding monocationic ionic liquids by two times. The investigation of the difference in the interaction of CO_2_ with the monocationic ILs and dicationic ILs was governed by the quantum chemical calculations [79]. Cao, et al. [80] synthesized four SILMs from imidazolium-based ILs and PVDF microporous membranes. The results of their study have shown that the synthesized SILMs have maintained an excellent CO_2_ absorption level during several cycles of absorption–desorption. Based on the surface-enhanced Raman scattering (SERS) spectra, a new peak was observed at 1274–1284 cm^−1^ which indicates that there is an absorbed CO_2_, and there are shifts in the vibration bands of the typical ILs which might be owed to some possible structures that are related to the CO_2_ absorption. In addition, the results of the authors’ research have shown that the SILMs have positively altered the mass transfer process and have resulted in an excellent absorption capacity of CO_2_. Furthermore, in the multiple absorption–desorption cycles, the SILMs have performed spectacularly and also the SERS is a feasible method that can be successfully conducted for the SILMs characterization [80]. Accordingly, it can be said that SILMs have successfully shown their capability of separating CO_2_, H_2_S, CH_4_, and N_2_ from different gas mixtures. Thus, SILMs have great potential in the gas separation processes used in the industrial field.

### 3.3. Ionic Liquid Polymeric Membranes (ILPMs)

At high transmembrane pressure, and high temperature, the stability of the SILMs is greatly affected by leaching of the ILs from the membrane support. Thus, limiting the SILMs application in the gas separation. In order to overcome these drawbacks, synthesizing a polymer/ILs composite membranes, at which the ILs are trapped within the narrow spaces of the individual polymer clusters or chains, has been shown to be a successful method in stabilizing the ILs in a polymeric matrix [81]. The usage of a polymer/ILs composite blend membrane offers chemical and physical interactions between the ILs and the polymer. The polymer/ILs composite membranes are usually referred to as ion gel membranes or polymer/IL gels. Most of the time, the polymer/ILs composite membranes are prepared under a controlled atmosphere by using a solvent casting method with solvent evaporation. Consequently, providing a mechanically, stable dense membrane. Polymeric ionic liquids (PILs) are generally synthesized by Ionic liquids (Ils) monomers with polymerizable groups such as styryl and vinyl groups.

**Figure 5:**
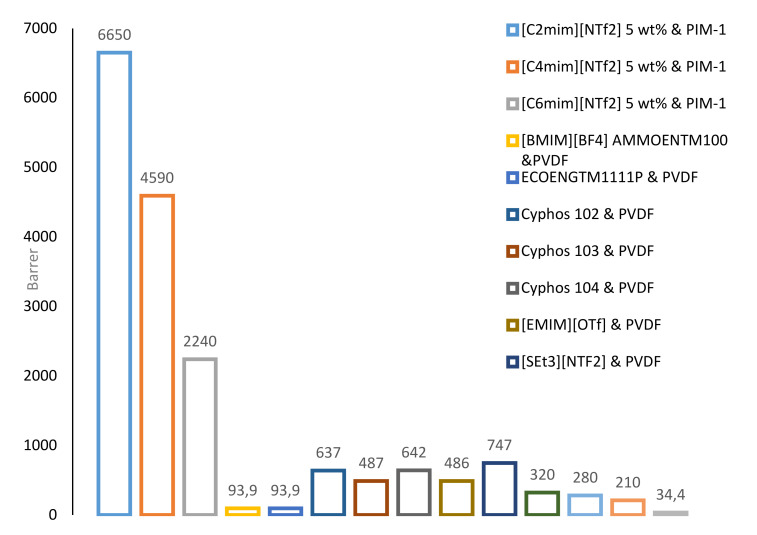
Permeability of CO_2_ through several ILPMs [88].

**Figure 6:**
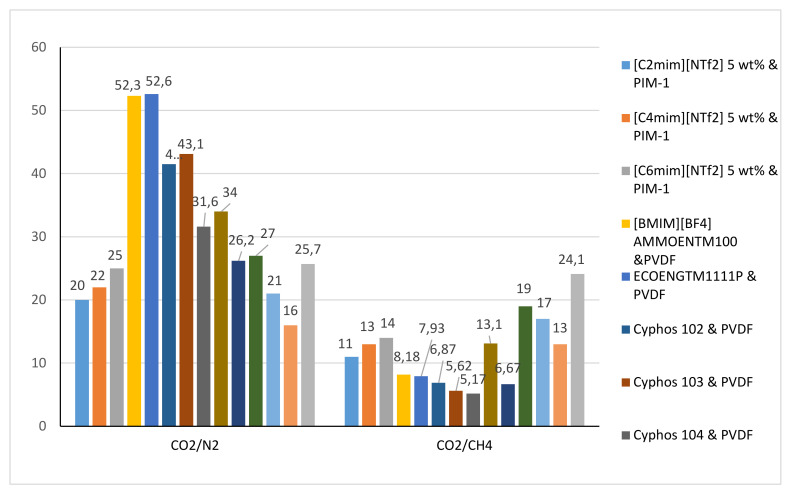
The selectivity of CO_2_/N_2_ and CO_2_/CH_4_ through several ILPMs [88].

The PILs have been the focus of many researchers recently as a result of their outstanding CO_2_ adsorption performance [82,83,84,85,86,87]. Figure 5 and Figure 6 below show the permeability of CO_2_ and the selectivity of CO_2_/N_2_ and CO_2_/CH_4_ through several ILPMs.

In Figure 5, the highest permeability of CO_2_ (6650 bar) of the ILPMs is the membrane with the ionic liquid [C_2_mim][NTf_2_] with five wt % and the polymer PIM-1. On the other side, the lowest permeability (34.4 bar) is related to [BMIM][NTf_2_] with the polymer PI.

Figure 6 shows that the highest and the lowest selectivity of CO_2_/N_2_ are 52.6 and 16 for ECOENG^TM^1111P and PVDF and [MtdFHim][NTf_2_] and PES, respectively. On the other hand, the highest and the lowest selectivity of CO2/CH4 are 24.1 and 5.17 for [BMIM][NTf_2_] and PI and Cyphos 104 and PVDF respectively.

A large number of studies have been made to study the applications of the ILPM in the gas separation process. Mannan, et al. [89] prepared ionic liquid polymeric membranes (ILPMs) at high concentrations of ionic liquid that combined polyethersulfone (PES) polymer with 1-ethyl-3-methyl imidazolium bis(trifluoromethylsulfonyl)imide ([EMIM][Tf_2_N]). The ionic liquid used in their study was (1-ethyl-3-methyl imidazolium bis(trifluoromethylsulfonyl) imide ([EMIM][Tf_2_N])) which has a high affinity for CO_2_ gas. The ionic liquid embedded within the membranes were examined at a temperature of 25 °C and a high-pressure range of 5–25 bar. Their study results show that as the IL concentration in ILPMs increases the permeability of CO_2_ and selectivity of CO_2_/CH_4_ increases simultaneously, as a result of high CO_2_ affinity in IL. Consequently, the ILPMs synthesized in their study are considered very promising membranes for the gas separation process of CO_2_ from CH_4_ gas at elevated IL concentrations and pressures [89].

In order to improve the film-forming property and gas permeability of poly(ionic liquid) membranes, a series of semi-interpenetrating polymer network (semi-IPN) membranes were successfully synthesized by incorporating linear polyvinyl acetate (PVAc) into a cross-linked poly(ionic liquid) (c-PIL) network by Zhang, et al. [90]. The resultant PVAc/c-PIL semi-IPN membranes in the authors’ research exhibited good film-forming property and various phase separation morphologies, from spherical, interconnected to co-continuous microstructure. Also, the formation of a semi-IPN structure endowed the membrane with improved mechanical properties and slightly depressed glass transition temperatures. From gas permeation measurement results, PVAc/c-PIL semi-IPN membranes demonstrated a gradually higher CO_2_ permeability coefficient with the increase of c-PIL content, while CO_2_/N_2_ permselectivity increased initially and then decreased slightly. The best permeability and permselectivity of the membrane reached in their study were 36.1 barrer and 59.6, respectively, when the c-PIL content was 50 wt %, which approached to the 2008 Robeson limit. Moreover, the permeability of CO_2_ in the PVAc/c-PIL-50 semi-IPN membrane increased with increasing temperature across their glass transition temperatures, while the permselectivity of CO_2_/N_2_ decreased [90]. Tomé, et al. [91] studied the effect of changing the compositions from pure ionic liquid to pure polymer membranes on the CO_2_ separation performance from flue gas and natural gas. In their research, a new polymeric ionic liquid composite membranes were synthesized that are based on poly([pyr11][NTf_2_]), poly(diallyldimethylammonium) bis(trifluoromethylsulfonyl)imide, and 1-butyl-1-methylpyrrolidinium bis(trifluoromethyl-sulfonyl)imide ([pyr14][NTf_2_]). The results of their study have shown that the permeability of CO_2_, CH_4_, and N_2_ in the IL is double the permeability values of the same three gases in the polymeric ionic liquid. Also, the composite membranes have resulted in the following; increase in the permeability values of all of the three gases, increase in the permselectivity for CO_2_/N_2_, decrease in the permselectivity for CO_2_/CH_4_ [91].

For the bulk removal of CO_2_ from flue gases and natural gas, a polymer/IL membrane (polyethersulfone/[EMIM][Tf_2_N]), a polyethersulfone membrane, were synthesized and their CO_2_/CH_4_ separation performance was measured [92]. The authors’ study results show that the permeability ofCO_2_ in the synthesized polymer/IL membrane has increased by blending the IL in the polymer matrix. This increase in permeability was owed by the authors to the high CO_2_ solubility in the [EMIM][Tf_2_N] IL. Also, the authors have mentioned that their synthesized ILPMs have successfully proved their capability of removing CO_2_ from flue gas and natural gas [92].

In order to overcome the high-temperature barrier that limits the use of SILMs in the gas separation, a thermally-stable composite ionic liquid and polymer membranes (CILPMs) have been synthesized Liang, et al. [93]. The CILPMs in their study were prepared by combining the Ionic liquid [C_4_mim][NTf_2_] with polybenzimidazole (PBI) and poly(pyromellitimide-co-4,4′-oxydianiline) (PMDA-ODA PI). The authors’ used a measurement rig to find the selectivities and permeabilities of the CILPMs for CO_2_, H_2_, CH_4_, N_2_, and CO over a wide range of temperatures and pressures. The results of their study have shown that their synthesized CILPMs had a high thermal and mechanical stability under the wide range of temperatures and pressures. Also, the change in temperature had enormously affected the selectivity and the permeability values of their membranes. On the other hand, changing the pressure had a small effect on the selectivity and the permeability values of their membranes. It was also found that incorporating the ionic liquid [C_4_mim][NTf_2_] into their membranes had significantly increased the CO_2_ permeation. Thus, the authors have mentioned that the CILPMs have very promising potential in the CO_2_ separation applications [93]. Tomé, et al. [94] studied the CO_2_, N_2_, and CH_4_ permeation properties of five distinct novel composite membranes based on polymeric ionic liquids (PILs). The PILs have different cation pendant units, which are; imidazolium, pyrrolidinium, pyridinium, cholinium, and ammonium, that are in combination with the same counter-anion ([NTf_2_]^−^). The results of their study have shown that the permeability of CO_2_ in the composite membranes is related to their respective diffusivities of CO_2_, which depends on the nature of the PIL polycation. The best permselectivities values of CO_2_ were found by the authors when tetra-alkyl ammonium-based PILs (ammonium, pyrrolidinium, and cholinium) were used. In addition, their results conclude that the PILs polycation backbones play a crucial role in the PIL–IL membranes design in order to obtain a high separation efficiency performance of CO_2_ [94]. Jansen, Friess, Clarizia, Schauer and Izák [81] prepared ionic liquid polymeric gel membranes by using the IL (1-ethyl-3-methylimidazolium bis(trifluoromethylsulfonyl)imide ([EMIM][TFSI]) in a poly(vinylidene fluoride-co-hexafluoro-propylene) (p(VDF-HFP)). Their membranes were synthesized via solvent casting from a solution in acetone. The results of their study have shown that there is an increase of permeability, especially for CO_2_ in the presence of [EMIM][TFSI]. Thus, they mentioned that the membranes synthesized can be used in the CO_2_ gas separation industry [81]. Mannan, et al. [95] proposed a method that can be used for predicting the CO_2_ gas permeability in an ionic liquid-polymeric membrane (ILPM). In their study, the methodology found was validated by using different ILPM systems. In addition, a satisfactory agreement was observed by the authors for both the experimental permeability and the predicted permeability by using the approach proposed [95].

### 3.4. Ionic Liquid Mixed Matrix Membranes (ILMMMs)

**Figure 7:**
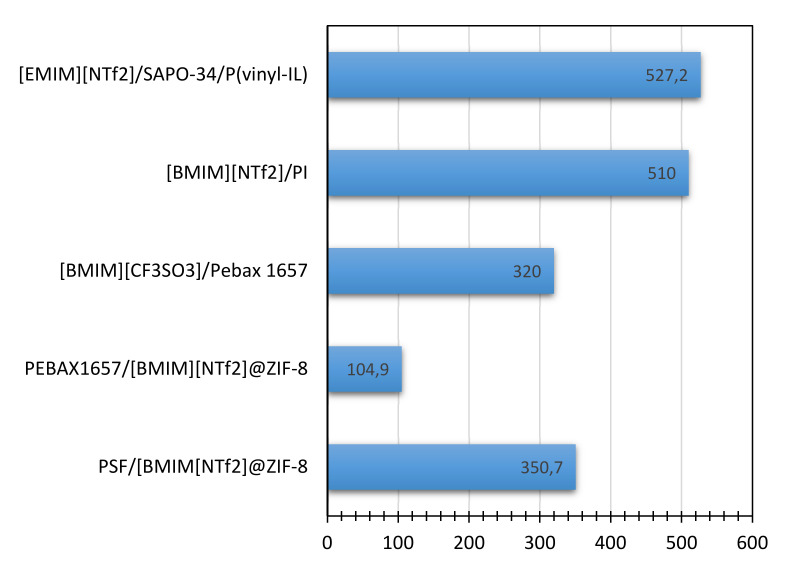
Permeability of CO_2_ through several ILMMMs [88].

**Figure 8:**
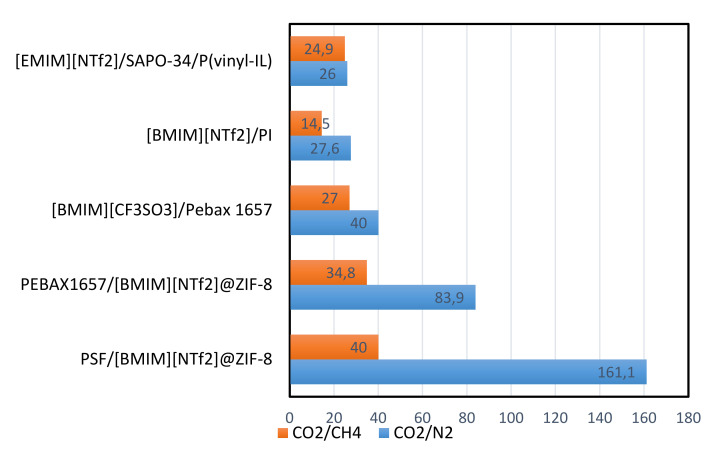
The selectivity of CO_2_/N_2_ and CO_2_/CH_4_ through several ILMMMs [88].

Nowadays, mixed matrix membranes (MMMs) have shown excellent progress in the gas separation processes. Many researchers are focusing their efforts on developing the MMMs in order to obtain better separation of gases [96]. The permeability of CO_2_ permeability and selectivity of CO_2_/N_2_ and CO_2_/CH_4_ for several ILMMMs are shown in Figure 7 and Figure 8 below.

One of the methods used in order to increase the gas separation efficiency of the MMMs is by the incorporation of the ionic liquids into the membrane forming Ionic liquid mixed matrix membrane (ILMMM). Bhattacharya and Mandal [97] examined the potentials of a MMM with an incorporated IL in the separation of CO_2_, or H_2_S, or both of the gases from their streams. In their study, a MMM was prepared by using the common ionic liquid 1-ethyl-3-methylimmidazolium-ethyl sulphate [EMIM][EtSO_4_] blended with a poly(ether-block-amide) (PEBA) elastomer. Many experiments were done by the authors for the pure gases (CO_2_/H_2_S/Air/CH_4_) under several IL concentrations and pressures. The results of their study have shown that the presence of the IL at five wt % has increased the H_2_S permeability reaching 540 bar.

The permeability values of the gases have been observed to follow the following descending order: H_2_S > CO_2_ > Air > CH_4_. Thus, the IL addition by the authors in the membrane matrix has positively altered and increased the acid gas permeability compared to the acid gases permeability of the pure PEBA membrane [97]. Ilyas, et al. [98] aimed to enhance the separation of CO_2_/N_2_ and CO_2_/CH_4_ through a mixed matrix membrane (MMM) and also improve the polymer-filler interaction. In their study, an acetate ion-based IL was used with and a (3-aminopropyl)trimethoxysilane in order to modify a zeolite 4A which acts as a filler in a polysulfone (PSF) membrane.

The permeability and selectivity data were obtained from several experiments made by the authors at different feed and temperatures conditions of the pure and mixed gases. The results of their study have shown that using the methoxy groups that contain cation and acetate anion based IL to modify the zeolites has enhanced the separation performance since the modified filler has improved the MMMs selectivity of 43% for CO_2_/N_2_ and 37% for CO_2_/CH_4_ at 30 wt % filler loadings as compared to the MMMs pristine filler [98]. Ahmad, et al. [99] revealed that the IL modified mixed matrix membrane has a better selectivity for CO_2_/N_2_ and CO_2_/CH_4_. A modification of a gas selective SAPO-34 zeolite was performed by using [EMIM][TF_2_N] IL before the incorporation of the zeolites into the polysulfone (PSF) asymmetric membrane which has been synthesized by using the phase inversion mechanism. The authors’ aimed to determine the immersion duration effects on the IL-zeolite functionality, the morphology of the membrane, and the PSF/SAPO-34 zeolite MMM gas separation properties. The energy dispersive X-ray analysis was conducted by the authors’ and has confirmed the incorporation of IL into the SAPO zeolite. The energy dispersive X-ray analysis and the scanning electron microscopy analysis have shown that there is an improvement in the polymer/filler interface morphology that has led to good dispersion of the IL-modified SAPO-34 particles for 6 h. Further results of their study have shown that the gas separation selectivity for 6 h of the membrane containing the IL-modified SAPO-34 has been greatly improved and enhanced by 486% for CO_2_/CH_4_ and 232% for CO_2_/N_2_ compared to the unmodified mixed matrix membrane MMM [99]. Mohshim, et al. [100] mentioned a huge improvement in the CO_2_/CH_4_ selectivity by using an ionically modified mixed matrix membrane over the pure mixed matrix membrane. In the authors’ study, the effect of incorporating IL into a flat sheet polyethersulfone-SAPO-34 MMM was investigated on the CO_2_ separation performance. The investigation was made by blending the IL [EMIM][Tf_2_N], at various concentrations, with the polymer-filler solution by using the solvent evaporation mechanism. The field emission scanning electron microscopy (FESEM) results have indicated that the membranes have a dense structure, and the thermogravimetric analysis (TGA) has shown that the membranes have a low polymer degradation temperature. The results of the authors’ experiments revealed that incorporation of IL into PES-SAPO-34 membrane has enhanced the CO_2_ separation. The authors’ mentioned that the ideal selectivity of CO_2_/CH_4_ obtained by using the highest IL concentration was 62.6 by comparing this selectivity value to the pure MMM selectivity value of CO_2_/CH_4_ which was 20.7 there is a large difference between the two values. This large difference between the two selectivity values shows that the ionic liquids have a great potential in enhancing the mixed matrix membranes separation performance of CO_2_ from CO_2_/CH_4_ mixture [100]. Nasir, et al. [101] have modified a filler surface (SAPO-34) by incorporating hexylamine (HA) and ethylenediamine (EDA). The authors of the study prepared four MMM by the incorporation of unmodified and modified fillers with the IL in a polyethersulfone (PES) matrix. The results of their study have shown that after the addition of the IL and modified SAPO-34 membranes were mechanically and thermally stable. Also, they mentioned that the synergetic effects of the ionic liquid IL and the modified filler had exhibited an excellent CO_2_/CH_4_ selectivity [101]. Ahmad, et al. [102] post-treated polysulfone (PSF)/silicoaluminophosphate (SAPO)-34 zeolite MMM by using three types of ionic liquid that have a high affinity for CO_2_. The results of their study have shown that the Mixed matrix membrane selectivity for CO_2_/N_2_ has been improved by 700% after the treatment with the ionic liquid solution 1-butyl-3-methylimidazolium tetrafluoroborate ([BMIM][BF_4_]) in methanol. In addition, when the ionic liquid was used, the CO_2_/N_2_ selectivity can be further increased to 44.9, and the CO_2_ permeance could be further increased to 7.19 GPU [102]. Huang, et al. [103] synthesized a facilitated transport MMMs by the incorporation of the ionic liquid functionalized graphene oxide (GO-IL) into the poly(ether-block-amide) (Pebax 1657). The 1-(3-aminopropyl)-3-methylimidazolium bromide IL was reacted with graphene oxide sheets, improving the solubility of CO_2_ and the gas selectivity of the MMMs. The results of their study have shown that there is an improvement greater than 90% in the CO_2_/N_2_ selectivity and approximately 50% in the CO_2_ permeability for the GO-IL MMMs compared when compared to the pure form of the Pebax membrane. Hence, the authors’ have mentioned that the incorporation of the ionic liquids has increased the performance of the MMMs [103]. Kamble, et al. [104] blended the IL 1-ethyl-3-methylimidazolium bis(trifluoromethylsulfonyl) imide with 2D materials, like hexagonal boron nitride (h-BN) and molybdenum disulfide (MOS_2_) in order to prepare polyether sulfone (PES) MMMs. The CO_2_, N_2_, and CH_4_ gas permeation along with the binary gas mixtures separation for CO_2_/CH_4_ and CO_2_/N_2_ were obtained by the authors for the pure and modified PES membranes. The results of their study have shown that the gas permeabilities were enhanced by approximately 15–20 times compared to the pure PES. Hence, the authors have mentioned that the usage of IL and 2D materials as a filler into the PES matrix has a significant enhancement in the gas separation/permeation properties of the PES and can be considered as a promising membrane for the CO_2_/N_2_ and CO_2_/CH_4_ separation [104].

One of the best single-gas separation performance in the literature was reported among the thermally rearranged polymers Singh, et al. [105]. In their study, cross-linked poly(room-temperature ionic liquids) (poly(RTIL)s), zeolite particles, and room-temperature ionic liquids (RTILs) were all combined to form mixed matrix membranes (MMMs). The MMMs prepared by the authors have exhibited a CO_2_ permeability of 260 ± 20 bar and selectivity of 90 ± 10 for CO_2_/CH_4_ [105]. Hudiono, et al. [106] synthesized a MMM containing three components: zeolite materials, polymerizable room-temperature ionic liquids (poly(RTIL)), and RTIL, and its transport characteristics were also investigated. The results of their study indicated that addition of ionic liquid increased the selectivity of the MMM [106]. Hudiono, et al. [107] reported that increasing the amount of room temperature ionic liquids (RTILs) in the MMMs increases the permeability of CO_2_ permeability [107].

### 3.5. Comparison Between SILMs, PILMs, and ILMMMs

Zia ul Mustafa, bin Mukhtar, Md Nordin, Mannan, Nasir and Fazil [25] compared between SILMs, PILMs, and ILMMMs by using a Robeson’s upper bound curves plot. On the Robeson’s upper bound curves, the general trade-off between selectivity and permeability for CO_2_/CH_4_ separation was shown. The results of the comparison made by the authors have shown that SILM and ILPMs have good permeability values. On the other hand, their selectivity values are just in touch with the commercial region. Moreover, the ILMMMs exhibits a lower permeability and a higher selectivity that is present above the commercial region compared to SILMs and ILPMs [25].

**Figure 9:**
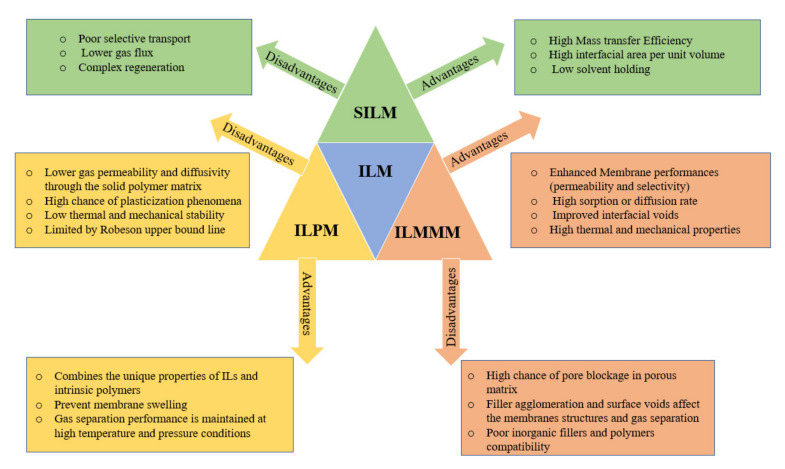
Summary of the advantages and disadvantages of SILM, ILPPM, and ILMMM.

Hence, it can be concluded from their comparison that for the gas separation processes that require high permeability of CO_2_/CH_4,_ SILMs, and ILPMs are the best ILMs to be used. However, for the processes that require high selectivity of CO_2_/CH_4,_ ILMMMs are the best ILMs to be used. Figure 9 above provides a summary of the advantages and disadvantages of SILM, ILPPM, and ILMMM.

## 4. Conclusions

The applications of the SILMs, ILPMs, and the ILMMMs in the gas separation process of CO_2_, CH_4_, N_2_, H_2_, or a mixture of these gases from various gas streams were analyzed in details through this paper. The effect of global warming has urged the separation of the greenhouse gases from the flue gas streams of the chemical industries. There are several techniques used in the gas separation of unwanted gases. This paper has focused on the membranes gas separation technique by using several classifications of ionic liquid membranes. A large number of articles were reviewed and analyzed. The results of the analysis show that SILMs, ILPMs, and the ILMMs are very promising membranes that have great potential in the gas separation processes. They offer a wide range of permeabilities and selectivities for CO_2_, CH_4_, N_2_, H_2_, or a mixture of these gases. In addition, a comparison was made based on the selectivity and permeability of SILMs, ILPMs, and ILMMMs for CO_2_/CH_4_ separation based on a Robeson’s upper bound curves. The results of the comparison have shown that SILMs and ILPMs have offered the highest permeability of CO_2_/CH_4,_ while ILMMMs have the highest selectivity for CO_2_/CH_4_. In the future, wise researchers should try to find ways in order to decrease the cost of ionic liquid membranes, decrease their energy consumption and increase their selectivity and permeability. By that, the ionic liquid membranes’ efficiency will be optimized.

## Figures and Tables

**Table 1:** Names and abbreviations of the principal Ionic liquid membranes and the primary gases discussed in this study.

Abbreviation	Name
ILM	Ionic Liquid Membrane
SILM	Supported Ionic Liquid Membrane
ILPM	Ionic Liquid Polymeric Membrane
ILMMM	Ionic Liquid Mixed Matrix Membrane
CILPMs	Composite Ionic Liquid and Polymer membranes
RTIL	Room Temperature Ionic Liquid
poly (RTIL)	Polymerizable room-temperature ionic liquids
CO_2_	Carbon Dioxide
N_2_	Nitrogen
NO_X_	Nitrogen Oxides
SO_X_	Sulphur Oxides
CH_4_	Methane
H_2_	Hydrogen
H_2_S	Hydrogen Sulphide
Greenhouse Gases	GHG

**Table 2:** Names and abbreviations of the ionic liquids used in this study.

Ionic Liquid	Ionic Liquid Name
[Ch][Lys]	Cholinium lysinate
[EMIM][BF_4_]	1-Ethyl-3-methylimidazolium tetrafluoroborate
[EMIM][DCA]	1-Ethyl-3-methylimidazolium dicyanamide
(P666n PF_6_)	Trihexylalkylphosphonium hexafluorophosphate
[EMIM][NTf_2_]	1-Ethyl-3-methylimidazolium bis (trifluoromethylsulfonyl) imide
[BMIM][BF_4_]	1-Butyl-3-methylimidazolium tetrafluoroborate
AMMOENG^TM^ 100	Quaternary ammonium salts
Ecoeng^TM^ 1111P	1,3-Dimethylimidazolium dimethylphosphate
Cyphos 102	Trihexyltetradecylphosphonium bromide
Cyphos 103	Trihexyltetradecylphosphonium decanoate
Cyphos 104	Trihexyltetradecylphosphonium bis(2,4,4-trimethylpentyl)phosphinate
[EMIM][CF_3_SO_3_]	1-Ethyl-3-methylimidazolium triflate
[SEt_3_][NTf_2_]	Triethylsulfonium bis(trifluoromethylsulfonyl) imide
[C_n_mim][NTf_2_] (*n* = 2, 4, 6)	(1-Alkyl-3-methylimidazolium bis(trifluoromethylsulfonyl) imide
[EMIM][OTf]	1-Ethyl-3-methylimidazolium trifluoromethanesulfonate
[MpFHim][NTf_2_]	1-Methyl-3-(3,3,4,4,4-pentylfluorohexyl) imidazolium bis(trifluoromethylsulfonyl)imide
[MnFHim][NTf_2_]	1-Methyl-3-(3,3,4,4,5,5,6,6,6-nonafluorohexyl) imidazolium bis(trifluoromethyl - sulfonyl)imide
[MtdFHim][NTf_2_]	1-Methyl-3-(3,3,4,4,5,5,6,6,7,7,8,8,8-tridecylfluorohexyl)imidazolium bis(trifluoromethyl-sulfonyl)imide
[BMIM][NTf_2_]	1-Butyl-3-methylimidazolium bis (trifluoromethylsulfonyl)imide
OMIM TCM	1-Methyl-3-octylimidazolium tricyanomethanide
[BMIM][Ac]	1-Butyl-3-methylimidazolium acetate
[EMIM][Ac]	1-Ethyl-3-methylimidazolium acetate
[Vbtma][Ac]	Vinylbenzyl trimethylammonium acetate
[BMIM][TfO]	1-Butyl-3-methylimidazolium trifluoromethanesulfonate
[BMIM][NTf_2_]	1-Butyl-3-methylimidazolium bis((trifluoromethyl)sulfonyl)imide
[BMIM][DCA]	1-Butyl-3-methylimidazolium dicyanamide
[BMIM][BF_4_]	1-Butyl-3-methylimidazolium tetrafluoroborate
[BMIM][PhO]	1-Butyl-3methylimidazolium phenolate
[pyr14][NTf_2_]	1-Butyl-1-methylpyrrolidinium bis(trifluoromethylsulfonyl)imide
[EMIM][TFSI]	1-Ethyl-3-methylimidazolium bis(trifluoromethylsulfonyl)imide
[EMIM][EtSO_4_]	1-Ethyl-3-methylimmidazolium-ethylsulphate
[BMIM][BETI]	1-Butyl-3-methylimidazolium bis(pentafluoroethylsulfonyl)imide
[BMIM][PF6]	1-Butyl-3-methylimidazolium hexafluorophosphate
[N(4)111][Tf_2_N]	Trimethyl(butyl)ammonium bis((trifluoromethyl)sulfonyl)imide
[N(6)111][Tf_2_N]	Trimethyl(hexyl)ammonium bis(trifluoromethyl)sulfonyl)imide
[N(10)111][Tf_2_N]	Trimethyl(decyl)ammonium bis(trifluoromethyl)sulfonyl)imide
[N(6)222][Tf_2_N]	Triethyl(hexyl)ammonium bis(trifluoromethyl)sulfonyl)imide
[N(1)444][Tf_2_N]	Tributyl(methyl)ammonium bis(trifluoromethyl)sulfonyl)imide
[N(1)888][Tf_2_N]	Trioctyl(methyl)ammonium bis(trifluoromethyl)sulfonyl)imide
[P(14)666][DCA]	Trihexyl(tetradecyl)phosphonium dicyanamide
[P(14)666][Tf_2_N]	Trihexyl(tetradecyl)phosphonium bis(trifluoromethylsulfonyl)imide
[P(2)444][DEP]	Ethyl(tributyl)phosphonium diethylphosphate
[P(14)444][DBS]	Tributyl(ethyl)phosphonium diethylphosphate
[EMIM][C(CN)_3_]	1-Ethyl-3-methylimidazolium tricyanomethide
[EMIM][N(CN)_2_]	1-Ethyl-3-methylimidazolium dicyanamide
[P666n][PF_6_]	Trihexylalkylphosphonium hexafluorophosphate

**Table 3:** Names and abbreviations of the polymers used in this study.

Polymer Abbreviation	Polymer Name
PIM^−1^	Polymers of intrinsic microporosity
PVDF	Polyvinylidene fluoride
PES	Poly(ether-sulfone) polymer
PI	Polyimide
PVAc	Polyvinyl acetate

**Table 4:** Two and three dimensional structures of the most used ionic liquids mentioned in this study.

Ionic Liquid	Two Dimensional (2D) Structure	Three Dimensional (3D) Structure
[EMIM][DCA]	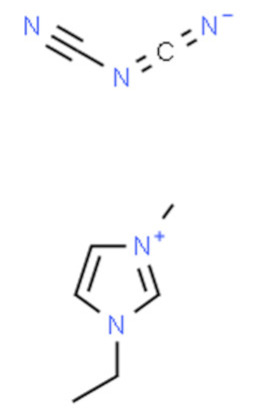	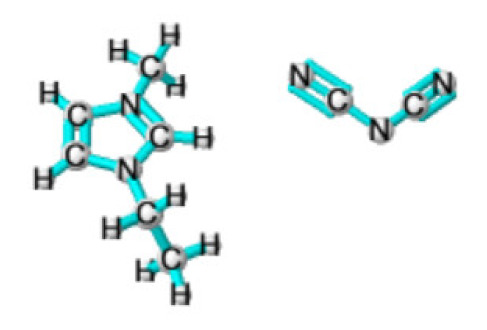
[EMIM][BF_4_]	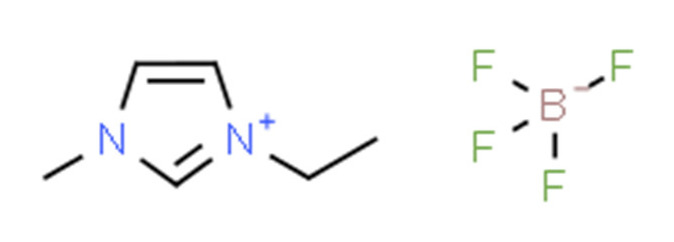	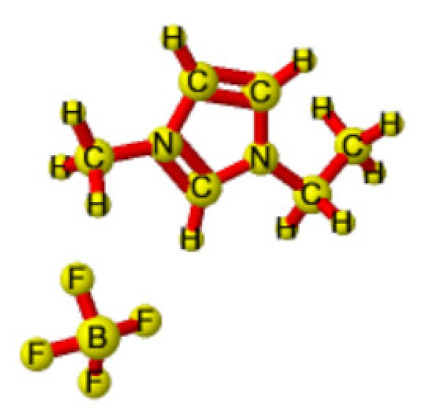
[EMIM][Ac]	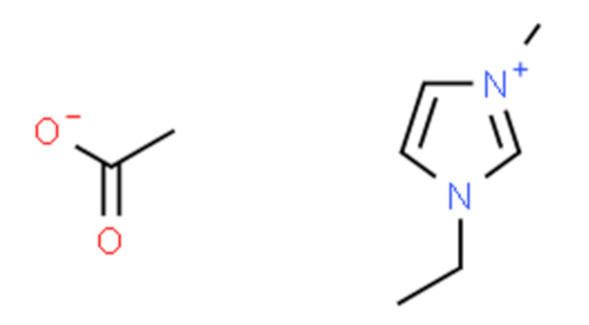	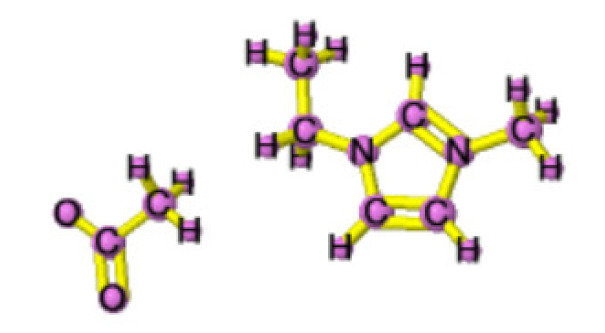
[EMIM][TFSI]	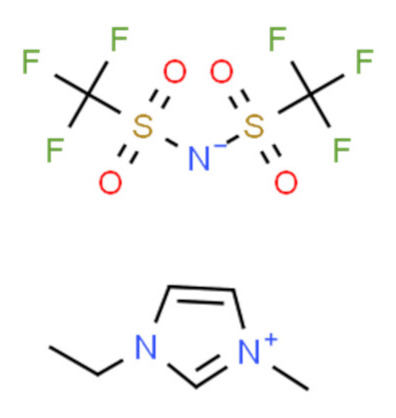	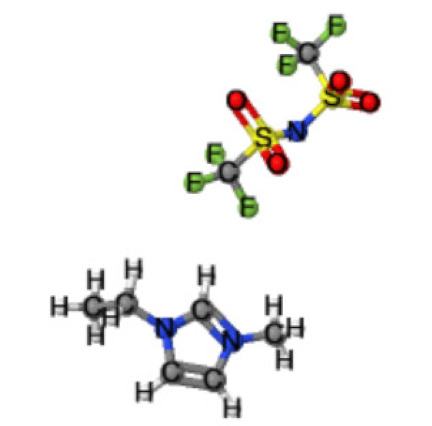
[BMIM][TFO]	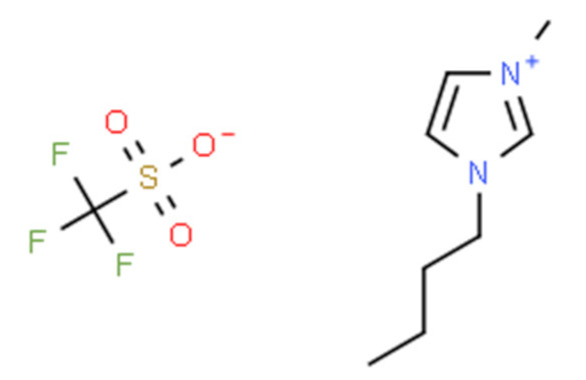	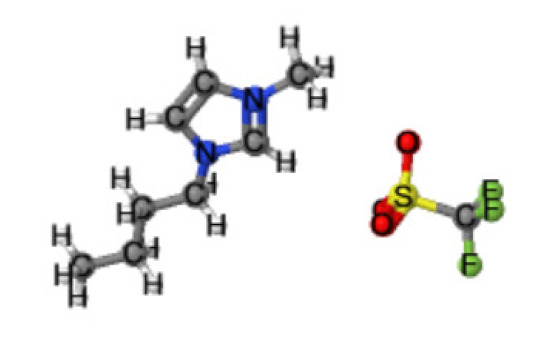
[BMIM][BF_4_]	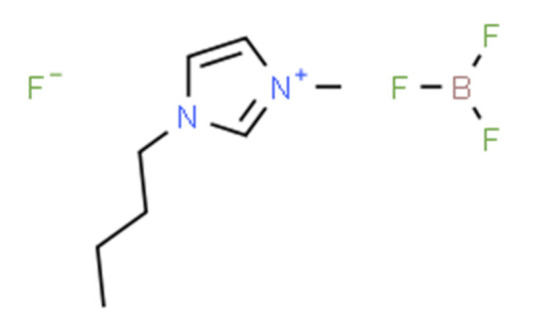	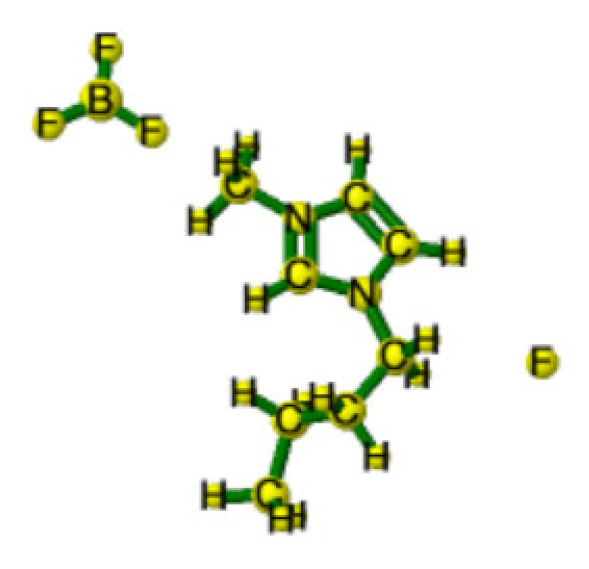
[BMIM][PF_6_]	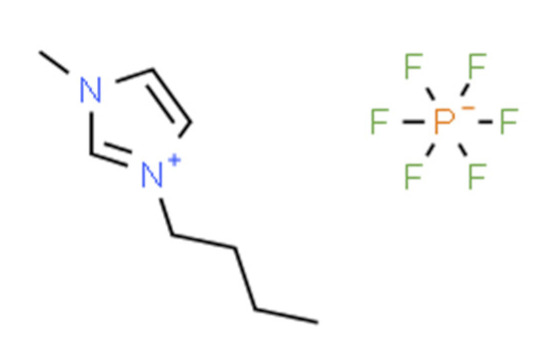	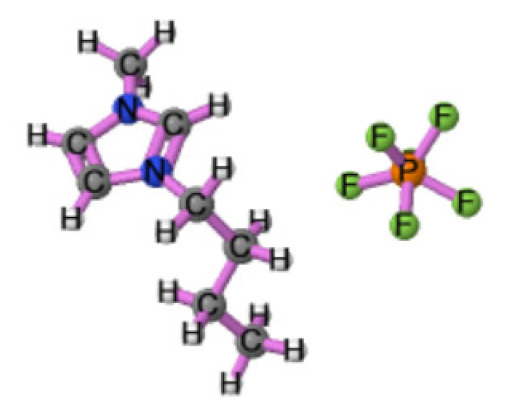
Cyphos 104	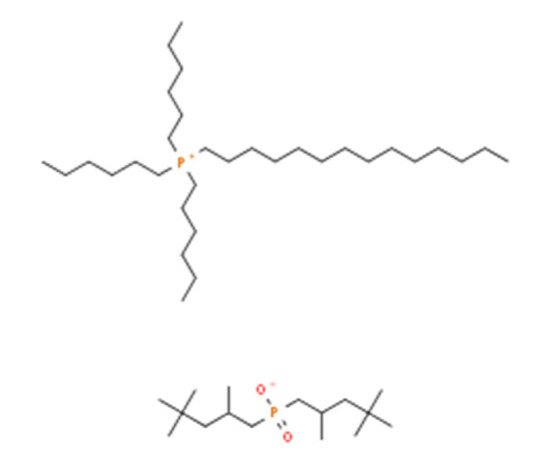	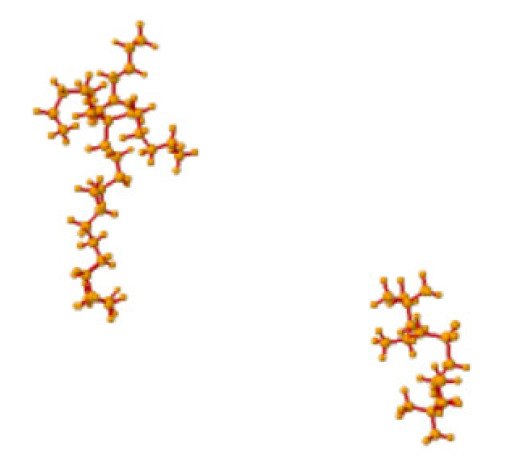
′[HMIM][BF_4_]	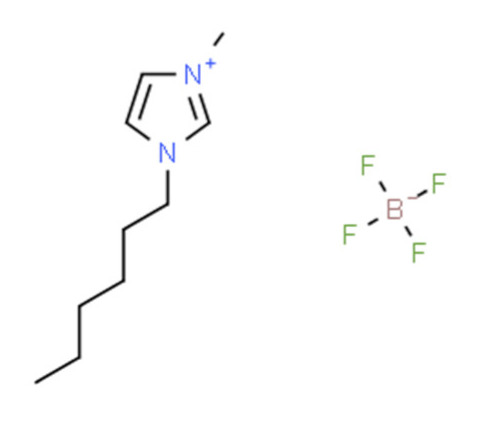	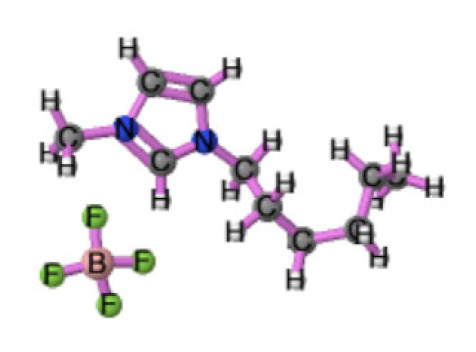

**Table 5:** Physicochemical properties of selected methylimidazolium ionic liquids sorted by the cation [39].

Cation	Anion	Formula	m.w g/mol	m.t. °C	Visc cP 25 °C	Visc cP 50 °C
MIM	Cl	C_4_H_7_ClN_2_	118.6	74	Solid	Solid
	NO_3_	C_4_H_7_N_3_O_3_	145.1	71	Solid	Solid
MMIM	Cl	C_5_H_9_ClN_2_	132.6	126	Solid	Solid
EMIM	Cl	C_6_H_11_ClN_2_	146.6	89	Solid	Solid
	SCN	C_7_H_11_N_3_S	169.3	−6	29	12
	Acetate NO_3_	C_8_H_14_N_2_O_2_ C_6_H_11_N_3_O_3_	170.2 173.2	−2 39	144 Solid	39
	N(CN)_2_Br	C_8_H_11_N_5_ C_6_H_11_BrN_2_	177.2 191.1	−18 75	16 Solid	9 Solid
	BF_4_	C_6_H_11_BF_4_N_2_	198	13	35	18
	Alaninate	C_9_H_17_N_3_O_2_	199.2	12	260	62
	C(CN)_3_CH_3_SO_3_	C_10_H_11_N_5_ C_7_H_14_N_2_O_3_S	201.2 206.3	−9 25	15 167	7 47
	CF_3_SO_3_HSO_4_	C_7_H_11_F_3_N_2_O_3_S C_6_H_12_N_2_O_4_S	260.2 208.2	−14 19	43 1600	20 360
	CH_3_SO_4_	C_7_H_14_N_2_O_4_S	222.3	−40	79	30
	C_2_SO_4_	C_8_H_16_N_2_O_4_S	236.3	−37	98	35
	C_4_SO_4_	C_10_H_20_N_2_O_4_S	264.3	−15	181	56
	C_6_SO_4_C_8_SO_4_	C_12_H_24_N_2_O_4_S C_14_H_28_N_2_O_4_S	292.4 320.5	−6 10	320 580	86,140
	I	C_6_H_11_IN_2_	238.1	78	Solid	Solid
	PF_6_	C_6_H_11_F_6_N_2_P	256.1	60	Solid	Solid
	AlCl_4_	C_6_H_11_AlCl_4_N_2_	280	6.5		
	TolSO_3_	C_13_H_18_N_2_O_3_S	282.4	50	Solid	240
	AsF_6_	C_6_H_11_AsF_6_N_2_	300.1	53	Solid	Solid
	NTf_2_	C_8_H_11_F_6_N_3_O_4_S_2_	391.3	−17	33	16
	N(SO2C2F5)2	C_10_H_11_F_10_N_3_O_4_S_2_	491.3	−1		
EMMIM	Br	C_7_H_13_BrN_2_	205.1	141	Solid	Solid
	NTf_2_	C_9_H_13_F_6_N_3_O_4_S_2_	405.3	25	Solid	
	N(SO2C2F5)2	C_11_H_13_F_10_N_3_O_4_S_2_	505.3	25	Solid	
PMIM	Cl	C_7_H_13_ClN_2_	160.6	62	Solid	Solid
	Br	C_7_H_13_BrN_2_	205.1	28	Solid	
	BF_4_I	C_7_H_13_BF_4_N_2_ C_7_H_13_IN_2_	212 252.1	−17 17	74	27
	PF_6_	C_7_H_13_F_6_N_2_P	270.2	38	Solid	
	NTf_2_	C_9_H_13_F_6_N_3_O_4_S_2_	405.3	15	46	19
BMIM	Cl	C_8_H_15_ClN_2_	174.5	41	Solid	150
	SCN	C_9_H_15_N_3_S	197.3	−6	51	19
	Acetate	C_10_H_18_N_2_O_2_	198.3	−1	430	67
	N(CN)_2_Br	C_10_H_15_N_5_ C_8_H_15_BrN_2_	205.3 219.1	−5 75	42 Solid	19 Solid
	BF_4_	C_8_H_15_BF_4_N_2_	226	−82	108	36
	C(CN)_3_HSO_4_	C_12_H_15_N_5_ C_8_H_16_N_2_O_4_S	229.3 236.3	−20 28	34 Solid	12
	ClO_4_	C_8_H_15_ClN_2_O_4_	238.7	8	180	57
	CH_3_SO_4_	C_9_H_18_N_2_O_4_S C_10_H_15_F_3_N_2_O_2_	250.3 252.2	−4 0	94 77	32 25
	Trifluoroacetate					
	PF_6_	C_8_H_15_F_6_N_2_P	284.2	11	270	74
	CF_3_SO_3_	C_9_H_15_F_3_N_2_O_3_S	288.3	14	80	31
	Cyclohexyl sulfamate	C_14_H_27_N_3_O_3_S	317.5	72.5	Solid	Solid
	FeCl_4_ C_8_SO_4_	C_8_H_15_C_l4_FeN_2_ C1_6_H_32_N_2_O_4_S	337 348.5	−12 24	41,870	18 152
	NTf_2_	C_10_H_15_F_6_N_3_O_4_S_2_	419.4	−5	42	19
Isobutylmim MMMMmim	NTf_2_ NTf_2_	C_10_H_15_F_6_N_3_O_4_S_2_ C_10_H_15_F_6_N_3_O_4_S_2_	419.4 419.4	−16 118	Solid	Solid
BMIM	Cl	C_9_H_17_ClN_2_	188.7	93	Solid	Solid
	BF_4_	C_9_H_17_BF_4_N_2_	240.1	32	Solid	31
	PF_6_	C_9_H_17_F_6_N_2_P	298.2	38	Solid	
	NTf_2_	C_11_H_17_F_6_N_3_O_4_S_2_	433.4	−6	350	34
Allylmim Benzmim	N(CN)_2_Cl	C_9_H_11_N_5_ C_11_H_13_ClN_2_	189.2 208.7	−20 14	20 3725	10 417
	CH_3_SO_4_	C_12_H_16_N_2_O_4_S	284.3	18	4500	327
	PF_6_	C_11_H_13_F_6_N_2_P	318.2	130	Solid	Solid
C5mim	PF_6_	C_9_H_17_F_6_N_2_P	298.2	16	380	97
	NTf_2_	C_11_H_17_F_6_N_3_O_4_S_2_	433.4	−9	58	22
HMIM	N(CN)_2_	C_12_H_19_N_5_	233.3	1	50	20
	BF_4_I	C_10_H_19_BF_4_N_2_ C_10_H_19_IN_2_	254.1 294.2	−82 30	200 Solid	58 246
	PF_6_ CF_3_SO_3_	C_10_H_19_F_6_N_2_P C_11_H_19_F_3_N_2_O_3_S	312.2 316.3	−61 25	480 Solid	120 47
	NTf_2_	C_12_H_19_F_6_N_3_O_4_S_2_	447.4	−6	70	27

**Table 6:** Methane and inorganic gas permeabilities and selectivities data at 30 °C.

Room Temperature Ionic Liquid (RTIL)	Permeabilities (Barrers) CO_2_	O_2_	N_2_	CH_4_	Selectivities		
					CO_2_/N_2_	CO_2_/CH_4_	O_2_/N_2_
*Imidazolium-RTILs* [EMIM][BF_4_]	968.5	32.1	21.8	43.7	44.5	22.2	1.5
[EMIM][TfO]	1171.4	60.8	28.9	63.2	40.5	18.5	2.1
[EMIM][Tf_2_N]	1702.4	143.5	73.6	139.2	23.1	12.2	1.9
[C_6_mim][Tf_2_N]	1135.8	121.6	73.9	134.0	15.4	8.5	1.6
[BMIM][BETI]	991.4	110.1	59.3	100.5	16.7	9.9	1.9
[BMIM][PF_6_]	544.3	36.2	21.2	40.8	25.6	13.3	1.7
*Ammonium-RTILs* [N(4)111][Tf_2_N]	830.5	97.6	40.5	63.1	20.5	13.2	2.4
[N(6)111][Tf_2_N]	943.2	97.0	46.2	102.1	20.4	9.2	2.1
[N(10)111][Tf_2_N]	800.4	106.0	53.9	105.0	14.8	7.6	2.0
[N(6)11(*i*-3)][Tf_2_N]	618.9	83.2	44.4	73.8	13.9	8.4	1.9
[N(10)11(*i*-3)][Tf_2_N]	632.8	96.0	67.5	114.3	9.4	5.5	1.4
[N(6)222][Tf_2_N]	630.3	67.9	31.9	64.5	19.8	9.8	2.1
[N(1)444][Tf_2_N]	523.9	74.4	35.3	68.5	14.8	7.6	2.1
[N(1)888][Tf_2_N]	619.4	116.0	54.9	138.9	11.3	4.5	2.1
*Phosphonium-RTILs* [P(14)666][Cl]-Source B	377.6	68.3	35.9	112.9	10.5	3.3	
[P(14)666][DCA]	513.7	87.1	36.2	107.6	14.2	4.8	
[P(14)666][Tf_2_N]	689.1	137.1	64.1	169.8	10.7	4.1	
[P(2)444][DEP]	453.4	71.5	31.0	90.1	14.6	5.0	
[P(14)444][DBS]	231.7	34.4	14.3	68.7	16.2	3.4	

## Supplementary Materials

The following are available online, Table S1: The median temperature variations in different locations. Figure S1: Worldwide search interest google trend of the Global Warming. Figure S2: Worldwide search interest google trend of the Greenhouse gases. Figure S3: Global CO_2_ concentration (ppm) in the atmosphere. Figure S4: CO_2_ gas emissions (tonnes) from gaseous processes in several Arab countries. Table S2: CO_2_ gas emissions (tonnes) from gaseous processes in several Arab countries. Figure S5: Worldwide search interest trend of the Carbon Dioxide (CO_2_) gas separation google. Figure S6: Global methane (CH_4_) gas concentration in the atmosphere (ppb). Figure S7: Worldwide membrane gas separation search interest. Figure S8: Membrane gas separation interest by region. Figure S9: Ionic Liquids google trend search by regions.
