# Supplementary material for: Key Applications and Potential Limitations of Ionic Liquid Membranes in the Gas Separation Process of CO2, CH4, N2, H2 or Mixtures of These Gases from Various Gas Streams
Source: Molecules. 2020 Sep 18;25(18):4274. doi: 10.3390/molecules25184274 (PMC7570638; doi:10.3390/molecules25184274)
Supplement: Supplementary file 1 [file molecules-25-04274-s001.pdf]

Table S1[1]. The median temperature variations in different locations

Year	Global (°C)	Northern Hemisphere) (°C)	Southern Hemisphere (°C)	Tropics (°C)
2000	0.294	0.404	0.184	0.17
2001	0.441	0.558	0.323	0.35
2002	0.496	0.593	0.398	0.469
2003	0.505	0.642	0.369	0.507
2004	0.447	0.604	0.289	0.43
2005	0.545	0.724	0.367	0.487
2006	0.506	0.676	0.336	0.449
2007	0.491	0.713	0.27	0.365
2008	0.395	0.569	0.22	0.274
2009	0.506	0.594	0.413	0.541
2010	0.56	0.74	0.379	0.535
2011	0.425	0.575	0.275	0.243
2012	0.47	0.627	0.312	0.346
2013	0.514	0.674	0.352	0.404
2014	0.579	0.779	0.381	0.516
2015	0.763	1.033	0.497	0.737
2016	0.797	1.064	0.531	0.763
2017	0.677	0.922	0.434	0.563
2018	0.597	0.789	0.405	0.473
2019	0.736	0.972	0.502	0.656

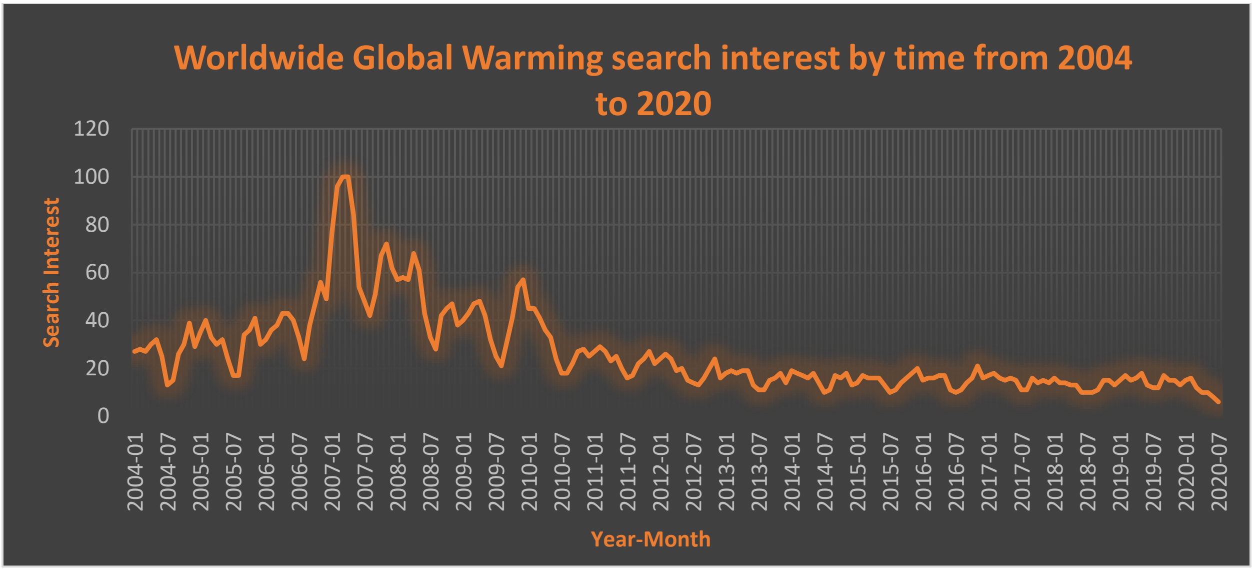

Figure S1. Worldwide search interest google trend of the Global Warming.

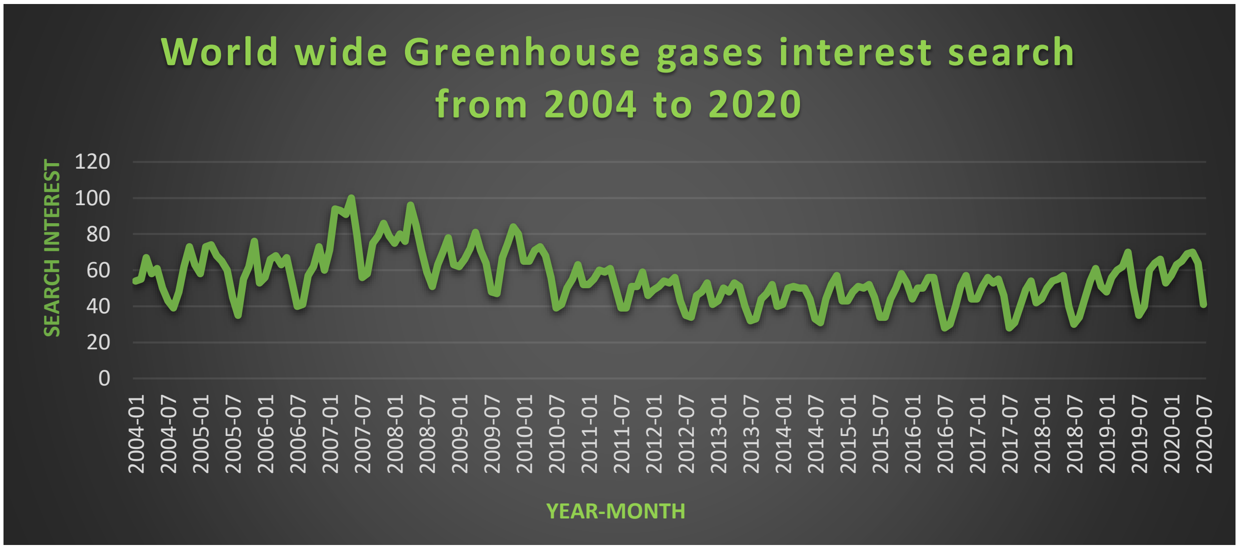

Figure S2. Worldwide search interest google trend of the Greenhouse gases.

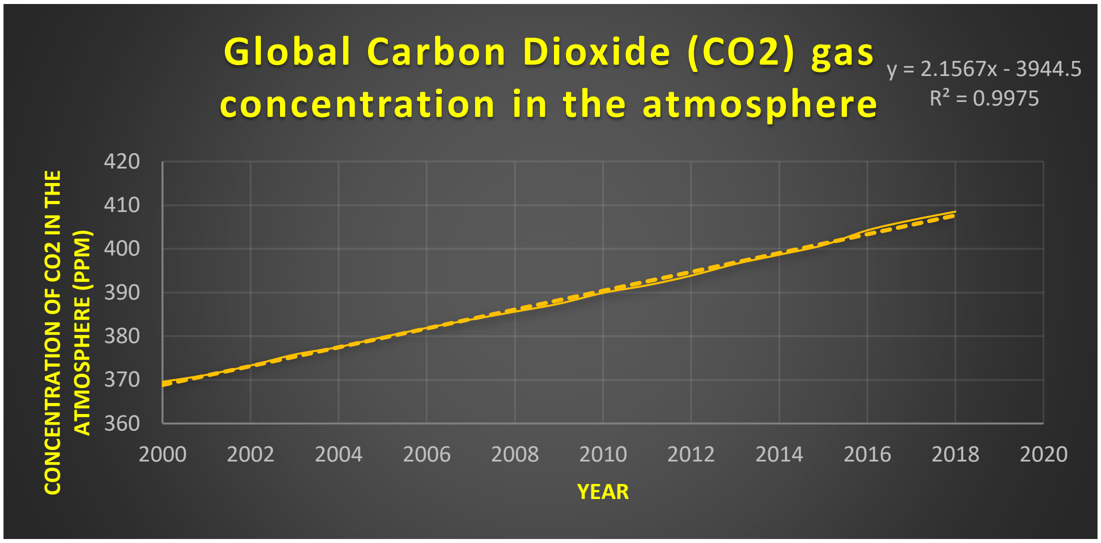

Figure S3[2]. Global CO_2 concentration (ppm) in the atmosphere.

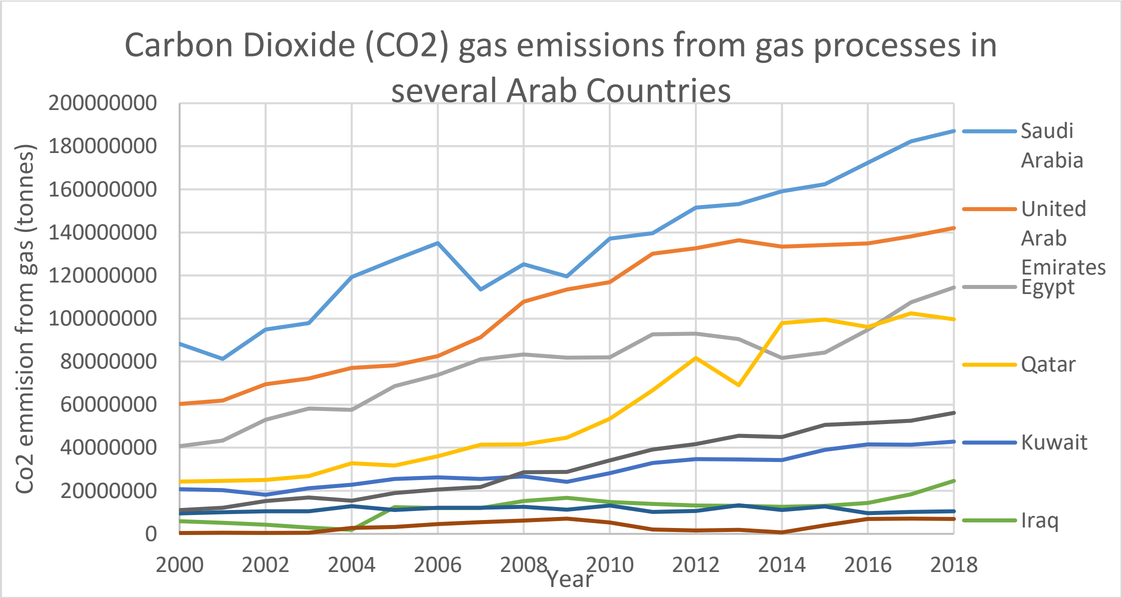

Figure S4. CO_2 gas emissions (tonnes) from gaseous processes in several Arab countries.

Table S2[3]. CO_2 gas emissions (tonnes) from gaseous processes in several Arab countries

		Carbon Dioxide (CO_2) gas emissions from gas processes (tonnes)							
Year	**Saudi Arabia**	**Emirates**	**Egypt**	**Qatar**	**Kuwait**	**Iraq**	**Libya**	**Jordan**	**Oman**
2000	88181500	60331400	40718000	24286100	20685500	5888000	9497100	443340	11028600
2001	81252900	61928900	43361000	24609600	20241200	5158900	10076000	472550	12133700
2002	94937900	69525100	52970400	24992100	18151500	4297900	10431400	428690	15314100
2003	97902100	72211300	58212200	26791200	21152300	2916500	10563300	487310	16817800
2004	119244900	77073400	57625100	32836800	22764400	1868600	12864300	2737000	15370500
2005	127254400	78226400	68621600	31814500	25527100	12439300	11038900	3165700	18891600
2006	135000100	82560900	73833300	36086700	26234200	11959300	12050900	4583700	20624700
2007	113477700	91295900	81128300	41466700	25539300	12028200	12106700	5506100	21716500
2008	125151200	1.08E+08	83310500	41564400	26619000	15249600	12575700	6236100	28691600
2009	119593000	1.13E+08	81787800	44730100	24101800	16700500	11234500	7060500	28732000
2010	137077600	1.17E+08	81947500	53395500	28132200	14817200	13196900	5235900	34108200
2011	139616700	1.3E+08	92680900	66681100	32857400	13908500	10167600	1996900	39114200
2012	151587000	1.33E+08	92919000	81670100	34731100	13153800	10709900	1509300	41636600
2013	153199200	1.36E+08	90398200	69087700	34566200	13058200	13271000	1868600	45632400
2014	159127500	1.33E+08	81701200	97916400	34281800	12633200	11105600	619220	44967300
2015	162344900	1.34E+08	84151100	99455600	39065600	12985500	12732400	4004800	50575000
2016	172307800	1.35E+08	94824300	96066400	41593700	14285900	9552700	6979900	51541500
2017	182301700	1.38E+08	1.07E+08	1.02E+08	41353000	18386400	10128100	7086600	52531600
2018	187089100	1.42E+08	1.14E+08	99599100	42829100	24613500	10558900	6883700	56177600

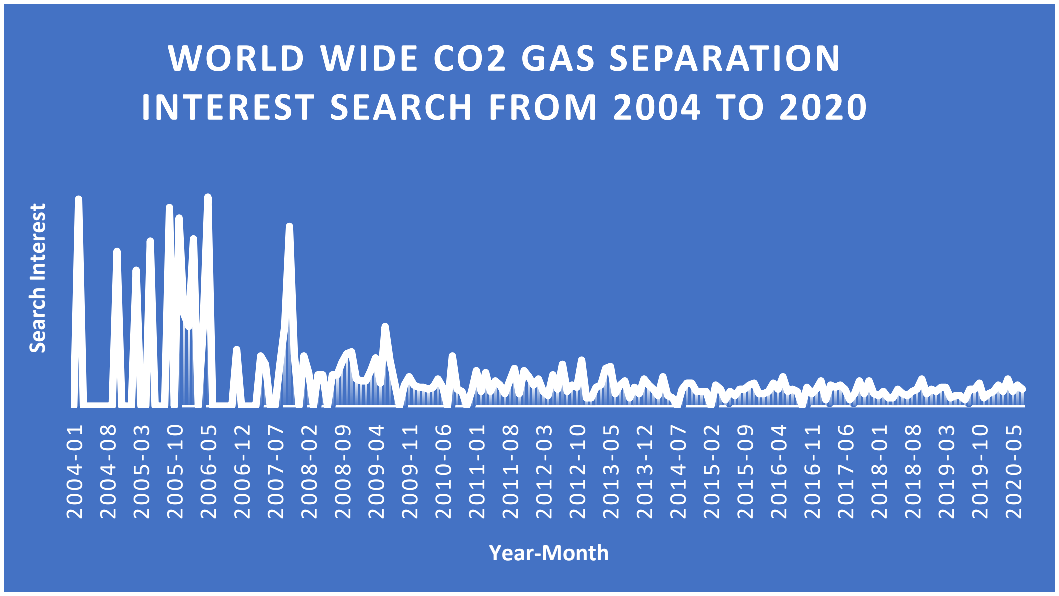

Figure S5. Worldwide search interest trend of the Carbon Dioxide (CO_2) gas separation in Google

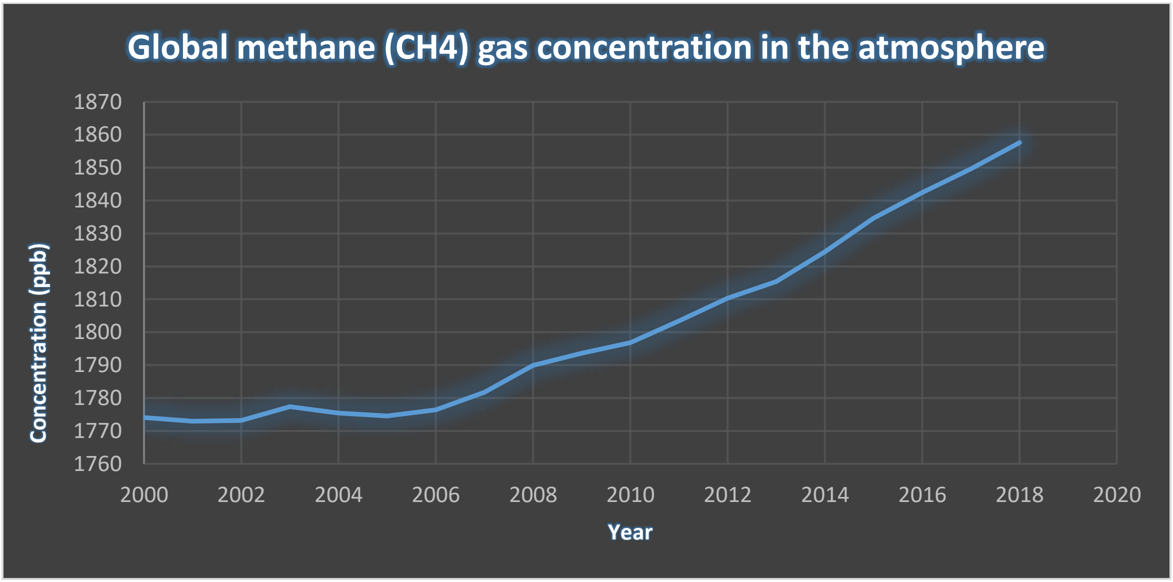

Figure S6[4]. Global methane (CH_4) gas concentration in the atmosphere (ppb).

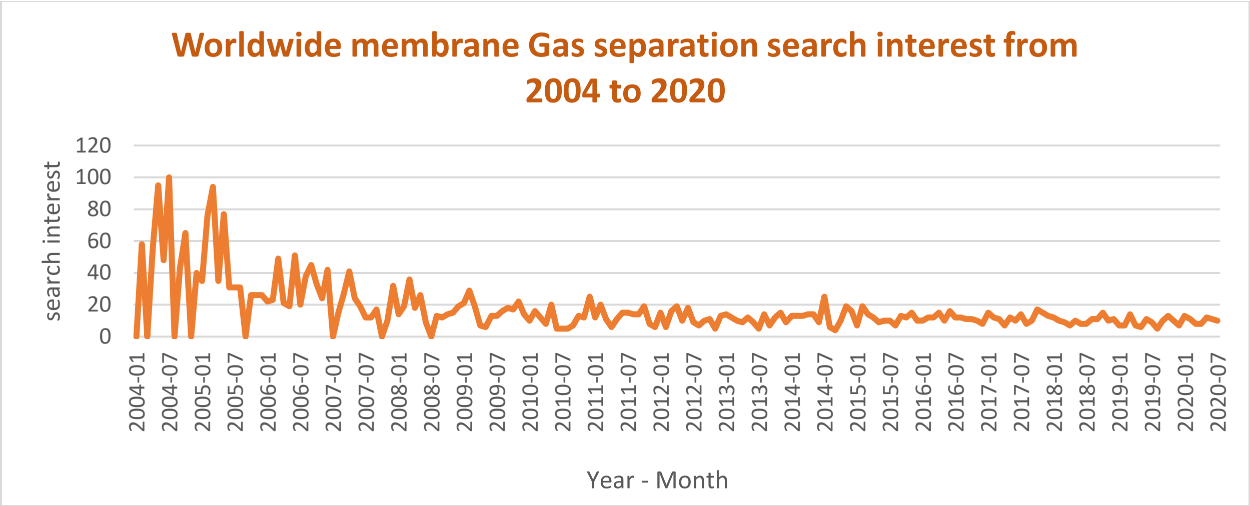

Figure S7. Worldwide membrane gas separation search interest

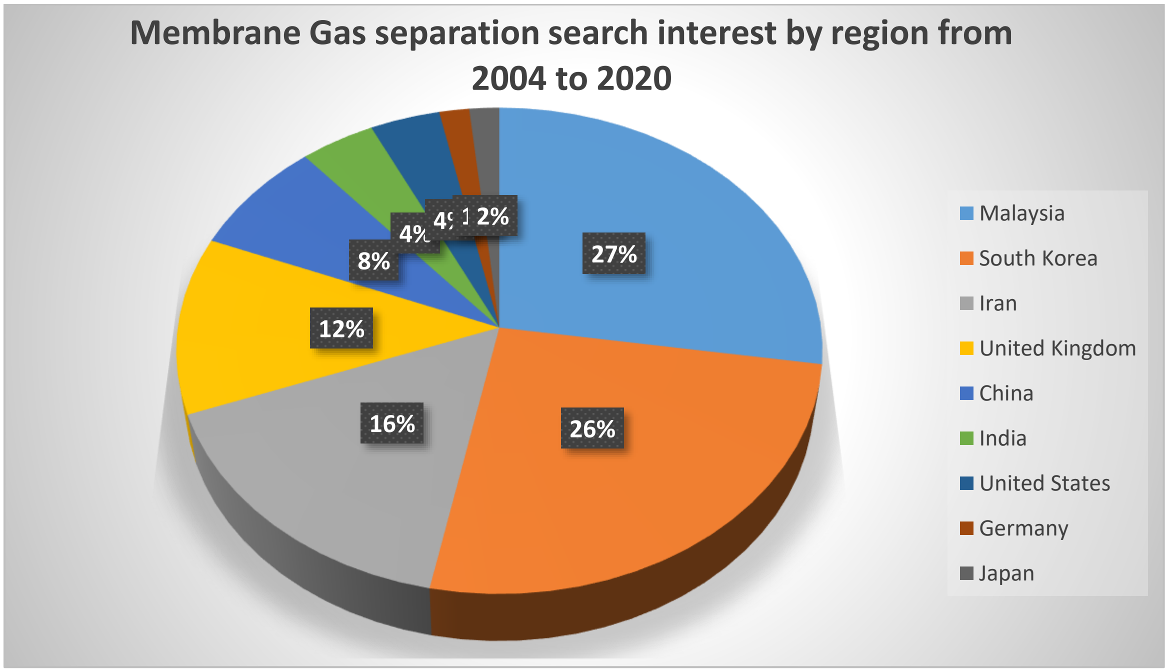

Figure S8. Membrane gas separation interest by region

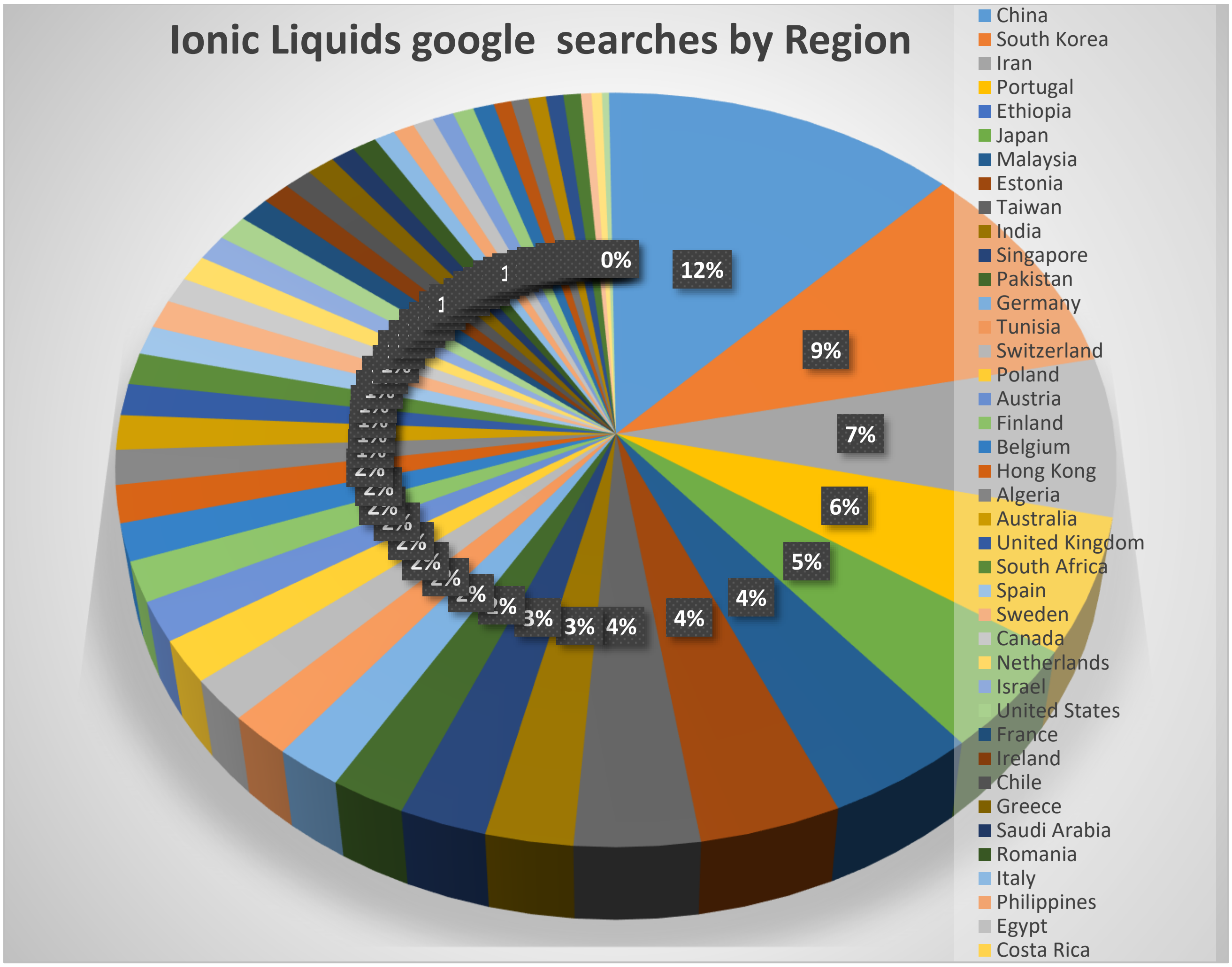

Figure S9. Ionic Liquids google trend search by regions

References

1. Morice, C.P.; Kennedy, J.J.; Rayner, N.A.; Jones, P.D. Quantifying uncertainties in global and regional temperature change using an ensemble of observational estimates: The HadCRUT4 data set. *Journal of Geophysical Research: Atmospheres* **2012**, *117*, doi:10.1029/2011JD017187.

2. Tans, P.; Ralph Keeling. Trends in Atmospheric Carbon Dioxide. Availabe online: https://www.esrl.noaa.gov/gmd/ccgg/trends/data.html (accessed on 29/August).

3. Friedlingstein, P.; Jones, M.W.; O'Sullivan, M.; Andrew, R.M.; Hauck, J.; Peters, G.P.; Peters, W.; Pongratz, J.; Sitch, S.; Le Quéré, C., et al. Global Carbon Budget 2019. *Earth Syst. Sci. Data* **2019**, *11*, 1783-1838, doi:10.5194/essd-11-1783-2019.

4. Dlugokencky, E. Trends in Atmospheric Methane. Availabe online: www.esrl.noaa.gov/gmd/ccgg/trends_ch4/ (accessed on 29/August).
